# Characterization of Aromatase Expression in the Adult Male and Female Mouse Brain. I. Coexistence with Oestrogen Receptors α and β, and Androgen Receptors

**DOI:** 10.1371/journal.pone.0090451

**Published:** 2014-03-19

**Authors:** Davor Stanić, Sydney Dubois, Hui Kheng Chua, Bruce Tonge, Nicole Rinehart, Malcolm K. Horne, Wah Chin Boon

**Affiliations:** 1 Systems Neurophysiology, The Florey Institute of Neuroscience and Mental Health, University of Melbourne, Parkville, Victoria, Australia; 2 Neurodegeneration, The Florey Institute of Neuroscience and Mental Health, University of Melbourne, Parkville, Victoria, Australia; 3 Department of Florey Neuroscience, University of Melbourne, Parkville, Victoria, Australia; 4 Départment de Biologie, Ecole Normale Supérieure de Lyon, Lyon, France; 5 Centre for Developmental Psychiatry & Psychology, Monash University, Clayton, Victoria, Australia; 6 Neurology Department, St Vincent's Hospital, Fitzroy, Victoria, Australia; 7 Department of Anatomy and Developmental Biology, Monash University, Clayton, Victoria, Australia; University of Otago, New Zealand

## Abstract

Aromatase catalyses the last step of oestrogen synthesis. There is growing evidence that local oestrogens influence many brain regions to modulate brain development and behaviour. We examined, by immunohistochemistry, the expression of aromatase in the adult male and female mouse brain, using mice in which enhanced green fluorescent protein (EGFP) is transcribed following the physiological activation of the *Cyp19A1* gene. EGFP-immunoreactive processes were distributed in many brain regions, including the bed nucleus of the stria terminalis, olfactory tubercle, medial amygdaloid nucleus and medial preoptic area, with the densest distributions of EGFP-positive cell bodies in the bed nucleus and medial amygdala. Differences between male and female mice were apparent, with the density of EGFP-positive cell bodies and fibres being lower in some brain regions of female mice, including the bed nucleus and medial amygdala. EGFP-positive cell bodies in the bed nucleus, lateral septum, medial amygdala and hypothalamus co-expressed oestrogen receptor (ER) α and β, or the androgen receptor (AR), although single-labelled EGFP-positive cells were also identified. Additionally, single-labelled ERα−, ERβ- or AR-positive cell bodies often appeared to be surrounded by EGFP-immunoreactive nerve fibres/terminals. The widespread distribution of EGFP-positive cell bodies and fibres suggests that aromatase signalling is common in the mouse brain, and that locally synthesised brain oestrogens could mediate biological effects by activating pre- and post-synaptic oestrogen α and β receptors, and androgen receptors. The higher number of EGFP-positive cells in male mice may indicate that the autocrine and paracrine effects of oestrogens are more prominent in males than females.

## Introduction

It has been known for nearly forty years that oestrogens are produced in the brain by the local aromatisation of testosterone [1], and these locally synthesised oestrogens [2] may modulate neuronal functions and provide neuroprotection (reviewed by [3]). Regions of the brain that express aromatase were initially identified by *in situ* hybridization (see [4], [5]) and measurements of aromatase activity. High levels of aromatase activity [6] and its mRNA [6]–[10] have been detected in the hypothalamus, preoptic nucleus, sexually dimorphic nucleus, bed nucleus of the striata terminalis (BST), hippocampal formation [2] and cerebellum [11].

While regions of the brain expressing aromatase are similar in both sexes, there are important differences in their levels of expression. Aromatase activity is four times higher in the hypothalamus of the postpubertal porcine male than female [12]. Aromatase transcript levels are also higher in the male rat amygdala, BST and medial septal nucleus than the corresponding nuclei in females [13], [14], although these differences are not present in some species, including sheep [15].

Examination of the distribution of aromatase expression and sex hormone receptors in brain circuitry would be advanced if the full repertoire of investigative tools, including immunohistochemistry and Western blotting could be used. This is the case with avian models in which specific antibodies were available for avian aromatase. As a result the distribution of aromatase in avian brains is well defined, and has coincided with advances in understanding the effects of brain-derived steroids on behaviour of bird models. Indeed, studies in songbird point to sexual dimorphic distribution of aromatase and more importantly, to synaptic synthesis of neuroactive steroids such as oestrogens mediating fast, perisynaptic membrane actions (refer to reviews by [16], [17]).

Unlike the avian models, immunohistochemical studies in rodents have provided inconsistent results that are at odds with results from studies using *in situ* hybridization or aromatase activity. For example, a polyclonal antibody raised against the rat aromatase sequence found little aromatase immunoreactivity in cell bodies within the BST, and medial basal hypothalamic and preoptic areas [18], whereas *in situ* hybridization [19], and other immunohistochemical studies, demonstrated high levels in these regions [20]. Of further concern is that Zhao et al. [21], using polyclonal antibodies raised against human placenta aromatase, found intense aromatase immunoreactivity in the male adult rat oval nucleus of the BST and lateral division of the central amygdaloid nucleus, but could not detect aromatase transcript by *in situ* hybridization in these same nuclei.

Evidence has accumulated that classical ERα can be anchored to the cell membrane through palmitoylation of ERα C447 and insertion to membrane caveoli [22], [23]. This membrane bound ERα can elicit rapid, non-genomic actions [22]–[24]. In the view that palmitoylation of oestrogen receptors is essential for neuronal membrane signalling [25] and brain oestrogens produced by brain aromatase are ‘neuroactive steroids’ that alter neuronal excitability and rapid signalling (reviewed by [26]), it is imperative to describe the distribution of the neurites of aromatase-positive neurones relative to oestrogen receptor expressing neurones in mouse - a common animal model used to study behaviour due to the ease of manipulating gene expression.

Immunohistochemistry depends on the sensitivity and selectivity of the antibody for its epitope, but it also has the advantage of identifying the specific cellular location of proteins, and allows for co-labelling with antibodies raised against aromatase, and oestrogen and androgen receptors. In this study we have circumvented the difficulties associated with antibodies raised against aromatase by using a transgenic mouse model in which transcription of enhanced green fluorescent protein (EGFP) occurs following the physiological activation of the *Cyp19A1* gene, and thus labelling the soma as well as the fibres of the aromatase expressing cells. We have also compared the distribution of oestrogen receptor α and β, and the androgen receptor, with the EGFP expression in the brain of this mouse.

## Results

### Distribution of EGFP-LI in the adult mouse brain

**Figure 1:**
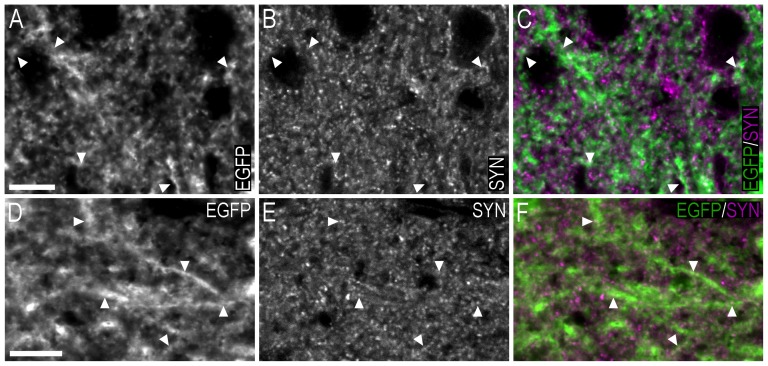
Double-immunofluorescence confocal micrographs showing the distribution of EGFP− and synaptophysin-LI in the bed nucleus of the stria terminalis and medial amygdaloid nucleus – posterodorsal part. (**A–C**) Single-channel confocal micrographs of double-fluorescence immunohistochemistry (C), displaying EGFP− (A), and synaptophysin-LI (B) in the bed nucleus of the stria terminalis. (**D–F**) Single-channel confocal micrographs of double-fluorescence immunohistochemistry (F), displaying EGFP− (D), and synaptophysin-LI (E) in the medial amygdaloid nucleus – posterodorsal part. In A–F, arrowheads point to double-labelled EGFP-ir nerve fibres/terminals and synaptophysin-LI. Scale bars: A = 10 µm, applies A–C; D = 10 µm, applies D–F.

**Figure 2:**
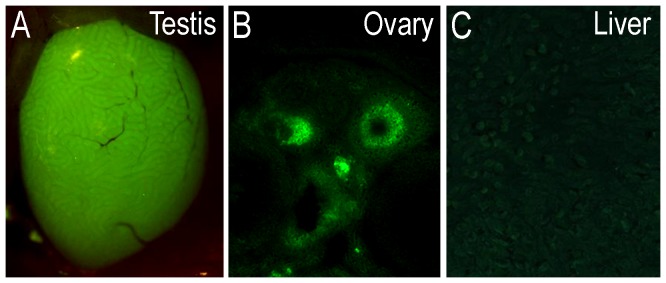
EGFP fluorescence in the testis, ovary and liver of adult transgenic mice. EGFP fluorescence in (**A**) the fresh testis and (**B**) granulosa layer in follicles of snap frozen ovary collected from adult transgenic animals. (**C**) No EGFP fluorescence was detected in the liver of adult transgenic mice.

The distribution of EGFP-ir cell bodies and processes was examined in brains of adult male and female mice, in which transcription of EGFP occurred following the physiological activation of the *Cyp19A1* gene. EGFP-LI was detected using the TSA+ immunohistochemical method, and a chicken antiserum raised against EGFP. Most EGFP+ cell bodies were round/ovoid in shape, and their size ranged from ∼10 to 15 µm in diameter. EGFP-LI was typically distributed in the cytoplasm, and was particularly intense towards the main axon/dendrite. The EGFP-LI appeared confluent and densely packed in many regions. However, under high magnification, and in particular using confocal microscopy, this fluorescence was ‘punctate’ in appearance and often colocalized with synaptophysin-LI (Fig. 1), thus was presumably confined to nerve endings. The expression pattern of the EGFP and aromatase has been confirmed by *in situ* hybridization [27], [28]. The relation of EGFP+ cell bodies to other neurone populations expressing ERα−, ERβ-, AR- or calbindin-LI is presented in merged colour micrographs of double-label immunohistochemistry, together with the corresponding black-and-white single channel micrographs. ERα−, ERβ-, and AR-LI were confined to the nucleus of cell bodies, with the diameter of immunoreactivity ranging from ∼8 to 12 µm. We will use abbreviations intermittently within subsections of the text to save space, and also to provide a ‘direct’ connection to the micrographs. For control purposes, EGFP fluorescence was detected in fresh testis and granulosa layer in follicles of snap frozen ovary collected from adult transgenic animals, while no EGFP fluorescence was detected in the liver of adult mice (Fig. 2A–C).

### Telencephalon

#### Basal forebrain

**Figure 3:**
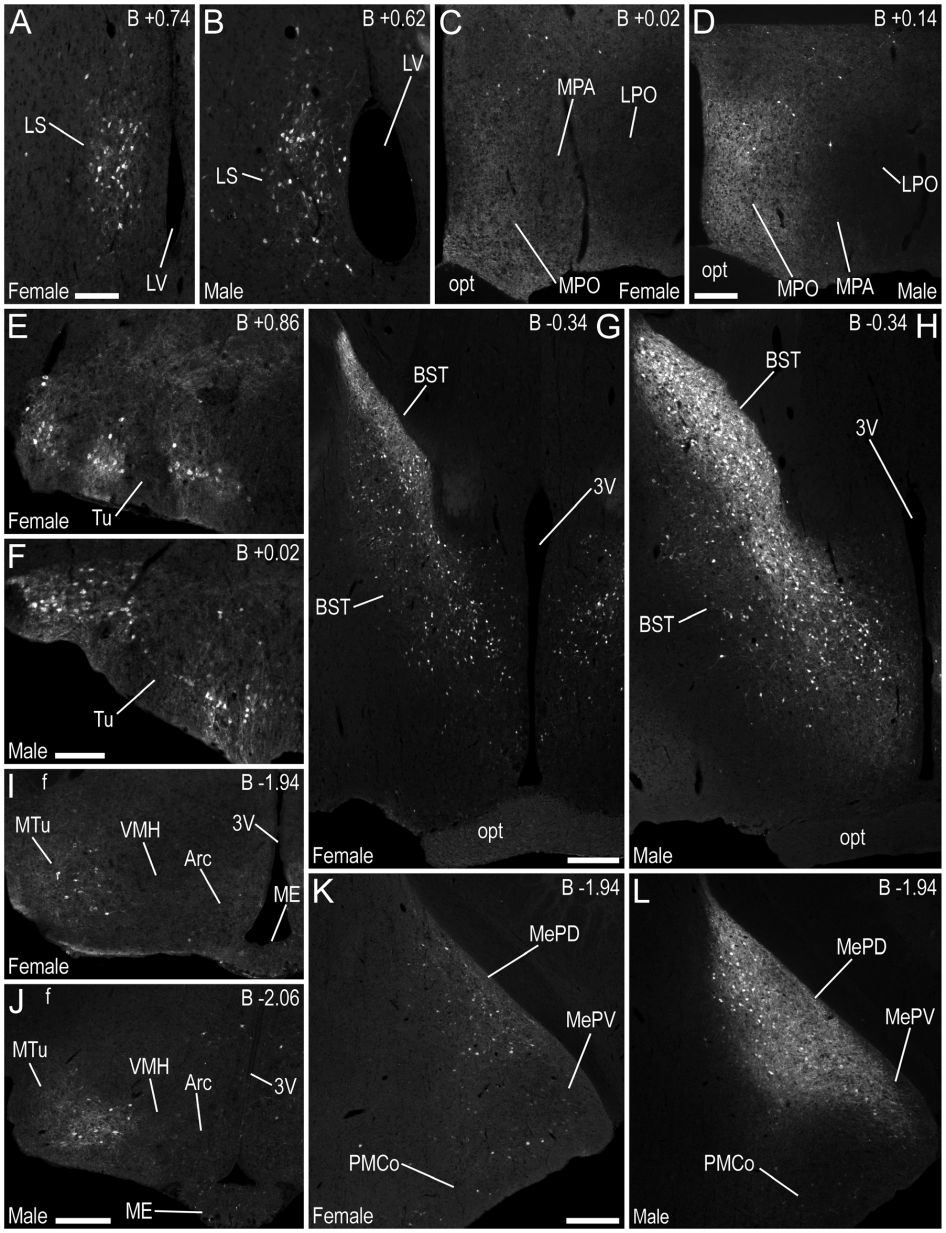
Immunofluorescence photomicrographs showing the regional distribution of EGFP-LI in coronal sections at different levels of the brain of female and male mice. EGFP-LI is shown at the level of the (**A, B**) lateral septal nucleus, (**C, D**) medial preoptic nucleus and medial preoptic area, (**E, F**) olfactory tubercle, (**G, H**) bed nucleus of the stria terminalis, (**I, J**) hypothalamus, including the arcuate, medial tuberal and ventromedial hypothalamic nuclei, and (**K, L**) medial amygdaloid nucleus. Distance from bregma (B) is indicated in the upper right corners, as shown in the Paxinos and Franklin atlas [73]. The stereotaxic co-ordinates are used only as a guide, and are given to allow the reader to refer to the Atlas mentioned above. Abbreviations: 3V, 3rd ventricle; Arc, hypothalamic arcuate nucleus; BST, bed nucleus of the stria terminalis; f, fornix; LPO, lateral preoptic area; LS, lateral septal nucleus; LV, lateral ventricle; ME, median eminence; MePD, medial amygdaloid nucleus, posterodorsal part; MePV, medial amygdaloid nucleus, posteroventral part; MPA, medial preoptic area; MPO, medial preoptic nucleus; MTu, medial tuberal nucleus; opt, optic tract; PMCo, posteromedial cortical amygdaloid nucleus; Tu, olfactory tubercle; VMH, ventromedial hypothalamic nucleus. Scale bars: A = 100 µm, applies A, B; D = 200 µm, applies C, D; F = 100 µm, applies E, F; G = 200 µm, applies G, H; J = 200 µm, applies I, J; K = 200 µm, applies K, L.

**Figure 4:**
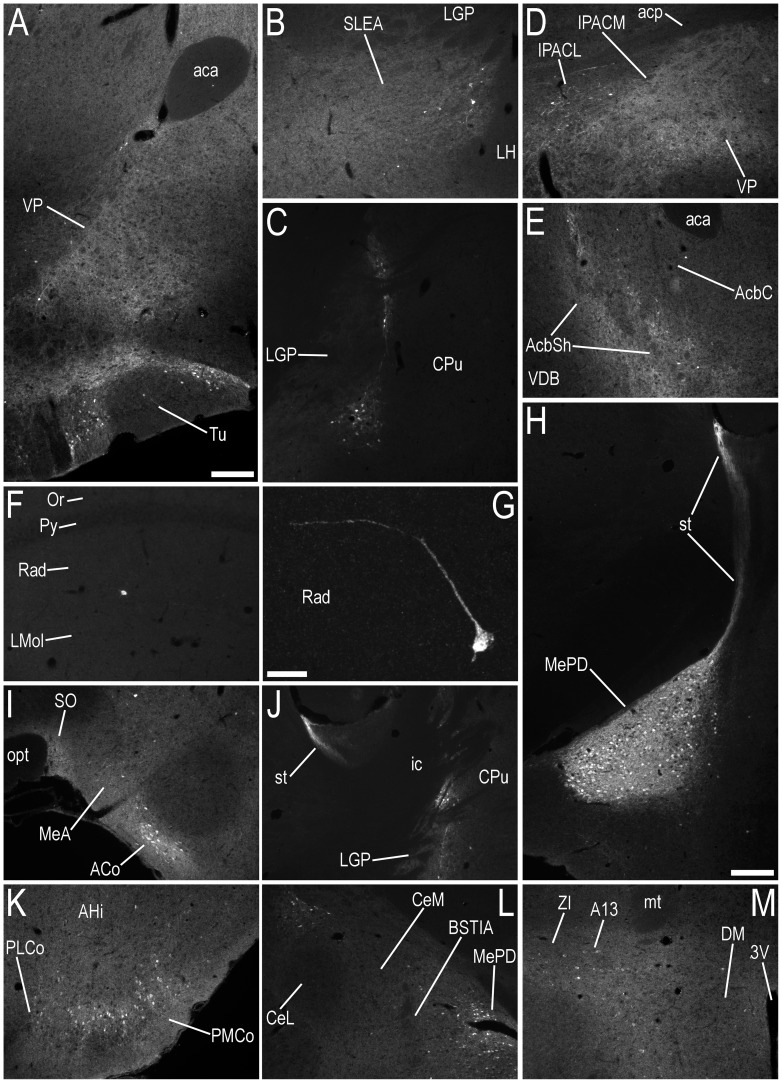
Immunofluorescence photomicrographs showing the regional distribution of EGFP-LI in coronal sections at different levels of the basal forebrain, hippocampal formation and amygdaloid complex of male or female mice. EGFP-LI is shown at the level of the (**A**) ventral pallidum and olfactory tubercle (Bregma, +0.38 mm), (**B**) sublenticular extended amygdala/substantia innominata (Bregma, −0.58 mm), (**C**) caudal, medial regions of the caudate putamen, bordering the globus pallidus (Bregma, −0.94 mm), (**D**) interstitial nucleus of the posterior limb of the anterior commissure – medial and lateral parts (Bregma, +0.14 mm), (**E**) accumbens nucleus – shell and core (Bregma, +1.10 mm), (**F, G**) stratum radiatum of hippocampal formation (Bregma, −1.94 mm), (**H**) medial amygdaloid nucleus – posterodorsal part and stria terminalis (Bregma, −1.94 mm), (**I**) anterior cortical amygdaloid nucleus, medial amygdaloid nucleus – anterior part and supraoptic nucleus (Bregma, −0.82 mm), (**J**) stria terminalis (Bregma, −1.06 mm), (**K**) posteromedial and posterolateral cortical amygdaloid nucleus (Bregma, −2.30 mm), (**L**) central amygdaloid nucleus – medial division, bed nucleus of the stria terminalis – intraamygdaloid division and medial amygdaloid nucleus – posterodorsal part (Bregma, −1.34 mm), and (**M**) the dorsomedial hypothalamic nucleus and zona incerta, including the A13 dopamine cell region (Bregma, −1.46 mm). The stereotaxic co-ordinates are used only as a guide, and are given to allow the reader to refer to the Paxinos and Franklin atlas [73] for orientation. Abbreviations: 3V, 3rd ventricle; A13, A13 dopamine cells; aca, anterior commissure, anterior part; AcbC, nucleus accumbens, core; AcbSh, nucleus accumbens, shell; ACo, anterior cortical amygdaloid nucleus; acp, anterior commissure, posterior; AHi, amygdalohippocampal area; BSTIA, bed nucleus of the stria terminalis, intraamygdaloid division; CeM, central amygdaloid nucleus, medial division; CeL, central amygdaloid nucleus, lateral division; CPu, caudate putamen (striatum); DM, dorsomedial hypothalamic nucleus; ic, internal capsule; IPACL, interstitial nucleus of the posterior limb of the anterior commissure, lateral part; IPACM, interstitial nucleus of the posterior limb of the anterior commissure, medial part; LGP, lateral globus pallidus; LH, lateral hypothalamic area; LMol, lacunosum moleculare layer of the hippocampus; MeA, medial amygdaloid nucleus, anterior part; MePD, medial amygdaloid nucleus, posterodorsal part; mt, mammillothalamic tract; opt, optic tract; Or, oriens layer of the hippocampus; PLCo, posterolateral cortical amygdaloid nucleus; PMCo, posteromedial cortical amygdaloid nucleus; Py, Pyramidal cell layer of the hippocampus; Rad, stratum radiatum of the hippocampus; SLEA/SI, sublenticular extended amygdala/substantia innominata; SO, supraoptic nucleus; st, stria terminalis; Tu, olfactory tubercle; VDB, nucleus of the vertical limb of the diagonal band; VP, ventral pallidum; ZI, zona incerta. Scale bars: A = 200 µm, applies A–F, I–M; G = 20 µm; H = 200 µm.

**Figure 5:**
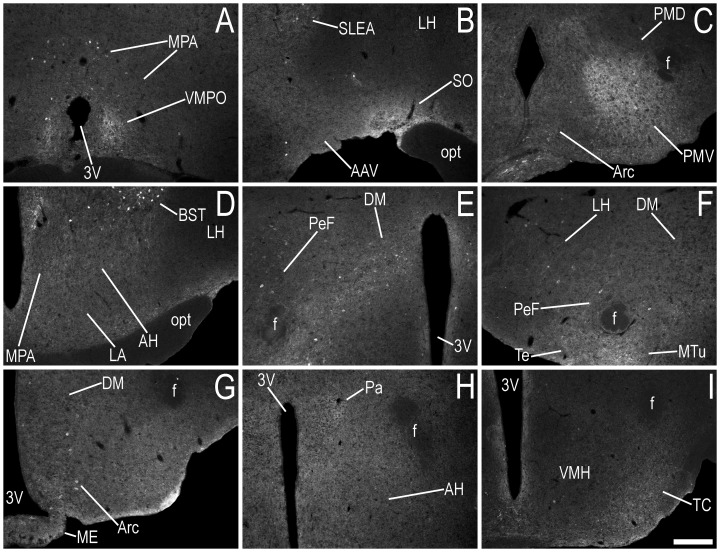
Immunofluorescence photomicrographs showing the regional distribution of EGFP-LI in coronal sections at different levels of the hypothalamus and amygdaloid complex of male or female mice. EGFP-LI is shown at the level of the (**A**) ventromedial preoptic nucleus (Bregma, +0.50 – +0.38 mm), (**B**) anterior amygdaloid area – ventral part and supraoptic nucleus (Bregma, −0.58 mm), (**C**) arcuate hypothalamic nucleus and premammillary nucleus – ventral and dorsal parts (Bregma, −2.46 mm), (**D**) anterior hypothalamic area and lateroanterior hypothalamic nucleus (Bregma, −0.58–−0.70 mm), (**E**) dorsomedial hypothalamic nucleus and perifornical area (Bregma, −1.82 mm), (**F**) lateral hypothalamic area, perifornical area and medial tuberal nucleus (Bregma, −1.94 mm), (**G**) arcuate hypothalamic nucleus, median eminence and dorsomedial hypothalamic nucleus (Bregma, −2.18 mm), (**H**) paraventricular hypothalamic nucleus and anterior hypothalamic area (Bregma, −0.82 mm), and (**I**) the tuber cinereum area (Bregma, −1.22 mm). The stereotaxic co-ordinates are used only as a guide, and are given to allow the reader to refer to the Paxinos and Franklin atlas [73] for orientation. Abbreviations; 3V, 3rd ventricle; AAV, anterior amygdaloid area, ventral part; AH, anterior hypothalamic area; Arc, hypothalamic arcuate nucleus; BST, bed nucleus of the stria terminalis; DM, dorsomedial hypothalamic nucleus; f, fornix; LA, lateroanterior hypothalamic nucleus; LH, lateral hypothalamic area; ME, median eminence; MPA, medial preoptic area; MTu, medial tuberal nucleus; opt, optic tract; Pa, paraventricular hypothalamic nucleus; PeF, perifornical area; PMD, premammillary nucleus, dorsal part; PMV, premammillary nucleus, ventral part; SLEA/SI, sublenticular extended amygdala/substantia innominata; SO, supraoptic nucleus; TC, tuber cinereum area; Te, terete hypothalamic nucleus; VMH, ventromedial hypothalamic nucleus; VMPO, ventromedial preoptic nucleus. Scale bars: I = 200 µm, applies A–I.

A very high density of strong EGFP-ir nerve fibres was found in the basal forebrain (Table 1), including the BST (Fig. 3G, 3H) and the stria terminalis (st) (Fig. 4H, 4J), with a high density of moderately strong EGFP-ir fibres in the olfactory tubercle (Tu) (Fig. 4A) and ventral pallidum (VP) (Fig. 4A, 4D). There was a moderate density of EGFP-ir fibres in the lateral septal nucleus (LS) (Fig. 3A, 3B), sublenticular extended amygdala (SLEA) (Figs. 4B, 5B), caudal, medial regions of the caudate putamen (CPu) (Fig. 4C, 4J), medial part of the interstitial nucleus of the posterior limb of the anterior commissure (IPACM) (Fig. 4D) and the accumbens nucleus shell (AcbSh) (Fig. 4E), with lower densities in the lateral part of the interstitial nucleus of the posterior limb of the anterior commissure (IPACL) (Fig. 4D), accumbens nucleus core (AcbC) (Fig. 4E) and the lateral globus pallidus (LGP) (Fig. 4C, 4J).

**Table 1:** Summary of GFP-like immunoreactivity in the cell bodies and nerve fibres throughout the adult male and female mouse brain.

STRUCTURE	Female	Male
	GFP^+^ cell bodies/(fibres)	GFP^+^ cell bodies/(fibres)
**TELENCEPHALON**		
***BASAL FOREBRAIN***		
Accumbens nucleus, core	- (+)	- (+)
Accumbens nucleus, shell	# (++)	# (++)
Bed nucleus of stria terminalis	### (++)	#### (++++)
Caudate putamen	# (−)	# (−)
Caudate putamen (caudal, medial)	## (++)	## (++)
Lateral globus pallidus	- (+)	- (+)
Horizontal limb of diagonal band	- (−)	- (−)
Interstitial nucleus of the posterior limb of the anterior commissure, medial part	# (++)	# (++)
Interstitial nucleus of the posterior limb of the anterior commissure, lateral part	# (+)	# (+)
Islands of Calleja	- (−)	- (−)
Lateral septal nucleus	## (++)	## (++)
Medial septal nucleus	- (−)	- (−)
Olfactory tubercle	### (+++)	### (+++)
Stria terminalis	- (++++)	- (++++)
Sublenticular extended amygdala/Substantia innominata	# (++)	# (++)
Ventral pallidum	# (+++)	# (+++)
Vertical limb of diagonal band	- (−)	- (−)
***AMYGDALOID COMPLEX***		
Amygdalohippocampal area	- (+)	- (+)
Anterior amygdaloid area, dorsal and ventral parts	- (+)	- (+)
Anterior cortical amygdaloid nucleus	## (++)	## (++)
Basolateral amygdaloid nucleus	- (−)	- (−)
Basomedial amygdaloid nucleus	- (−)	- (−)
Bed nucleus of stria terminalis, intraamygdaloid division	# (+)	# (+)
Central amygdaloid nucleus, medial division	# (+)	# (+)
Medial amygdaloid nucleus, anterior dorsal and anteroventral parts	# (++)	# (++)
Medial amygdaloid nucleus, anterior part	# (++)	# (++)
Medial amygdaloid nucleus, posterodorsal and posteroventral parts	### (+++)	#### (++++)
Posterolateral cortical amygdaloid nucleus	# (+)	# (+)
Posteromedial cortical amygdaloid nucleus	## (++)	## (++)
***CEREBRAL CORTEX***		
Cingulate cortex	# (−)	# (−)
Secondary motor cortex	# (−)	# (−)
***HIPPOCAMPAL FORMATION***		
CA1, CA2 and CA3	- (−)	- (−)
Dentate gyrus	- (−)	- (−)
Lacunosum moleculare layer	- (−)	- (−)
Stratum oriens	- (−)	- (−)
Stratum radiatum	# (−)	# (−)
**DIENCEPHALON**		
***HYPOTHALAMUS***		
Anterior hypothalamic area	# (+)	# (++)
Arcuate hypothalamic nucleus	# (+)	# (+)
Dorsomedial hypothalamic nucleus	## (++)	## (++)
Lateral hypothalamic area	# (+)	# (+)
Lateral preoptic area	- (−)	- (−)
Medial preoptic area	# (++)	## (+++)
Medial preoptic nucleus	# (++)	## (++++)
Medial tuberal nucleus	# (++)	# (+++)
Paraventricular hypothalamic nucleus	# (+)	# (+)
Perifornical area	# (++)	# (++)
Posterior hypothalamic area	- (−)	- (−)
Premammillary nucleus, dorsal part	- (+)	- (+)
Premammillary nucleus, ventral part	- (+++)	- (+++)
Suprachiasmatic nucleus	- (−)	- (−)
Supraoptic nucleus	- (+++)	- (+++)
Terete hypothalamic nucleus	# (++)	# (+++)
Tuber cinereum area	# (+)	# (+)
Ventromedial hypothalamic nucleus	- (−)	- (−)
Ventromedial preoptic nucleus	# (+)	# (++)
***THALAMUS***		
Zona incerta (A13)	# (+)	# (+)

(+) low, (++) moderate, (+++) high, or (++++) very high density/intensity of GFP-LI in fibres. Density of GFP-immunoreactive/positive (+) cell bodies is indicated as (#) sparse, (##) low, (###) medium, or (####) high.

Several groups of EGFP+ cell bodies were located within the LS (Fig. 3A, 3B), BST (Fig. 3G, 3H), Tu (Figs. 3E, 3F, 4A), some areas of the VP (Fig. 4A), and caudal, medial regions of the CPu, bordering the globus pallidus (Fig. 4C, 4J), as well as the AcbSh (Fig. 4E), IPAC (Fig. 4D), and SLEA (Figs. 4B, 5B) (Table 1).

The BST of male mice contained a greater density of EGFP-ir nerve fibres, and the number of EGFP+ cell bodies in the BST of female mice was 63% lower than the number estimated in male mice (Figs. 3H c.f. 3G, 6A; Table 2). In other regions of the basal forebrain, including the LS or Tu, the density/intensity of EGFP-ir fibres and EGFP+ cell bodies appeared similar in male and female mice (Fig. 3A c.f. 3B, 3E c.f. 3F, Table 1).

**Figure 6:**
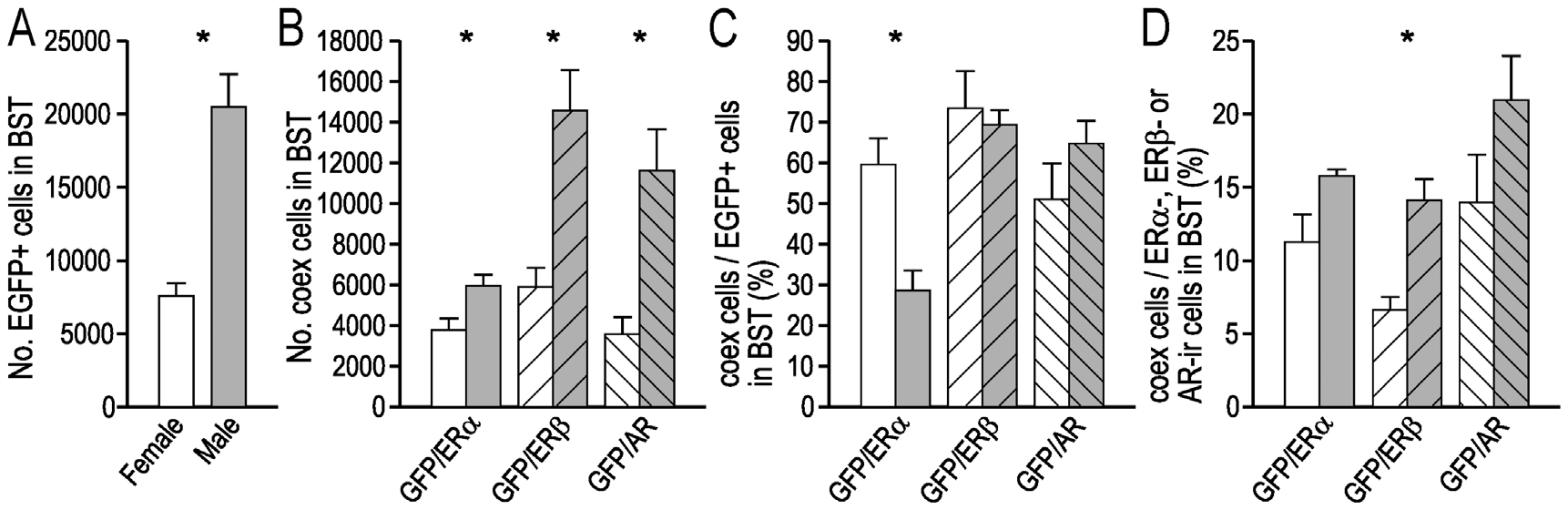
Quantification of EGFP+, ERα−, ERβ- and AR-ir cell bodies in the female and male BST. (**A**) Estimated number of EGFP+ cell bodies in the female and male BST (n = 5 per group). (**B**) Estimated number of EGFP+ cell bodies in the BST that coexpressed ERα, ERβ or AR-LI (n = 3 per group). (**C**) The number of EGFP+ cell bodies in the BST coexpressing ERα, ERβ or AR-LI, expressed as a percentage of the estimated number of EGFP+ cell bodies in the BST (n = 3 per group). (**D**) The number of EGFP+ cell bodies in the BST coexpressing ERα, ERβ or AR-LI, expressed as a percentage of the estimated number of ERα, ERβ or AR-ir cell bodies in the BST (n = 3 per group). In A–D, white bars = female mice, grey bars = male mice, * corresponds to *P*<0.05 (student *t*-test).

**Table 2:** Estimated number of EGFP+, ERα-ir, ERβ-ir and AR-ir cell bodies in the BST (mean ± SEM).

BST			
	Female	Male	*P*≤0.05
No. EGFP+ cells	7570±875 (n = 5)	20477±2239 (n = 5)	Yes
No. ERα-ir cells	34230±3880 (n = 3)	37940±4311 (n = 3)	No
No. ERβ-ir cells	89320±8592 (n = 3)	103000±6734 (n = 3)	No
No. AR-ir cells	25550±1152 (n = 3)	55860±659 (n = 3)	Yes
No. EGFP+/ERα-ir coexpressing cells	3780±556 (n = 3)	5950±547 (n = 3)	Yes
No. EGFP+/ERβ-ir coexpressing cells	5880±962 (n = 3)	14560±1993 (n = 3)	Yes
No. EGFP+/AR-ir coexpressing cells	3570±849 (n = 3)	11620±2019 (n = 3)	Yes
Proportion EGFP+ cells coexpressing ERα-ir	60±6% (n = 3)	29±5% (n = 3)	Yes
Proportion EGFP+ cells coexpressing ERβ-ir	73±9% (n = 3)	69±4% (n = 3)	No
Proportion EGFP+ cells coexpressing AR-ir	51±8% (n = 3)	65±6% (n = 3)	No
Proportion ERα-ir cells coexpressing EGFP	11±2% (n = 3)	16±0.4% (n = 3)	No
Proportion ERβ-ir cells coexpressing EGFP	7±1% (n = 3)	14±1% (n = 3)	Yes
Proportion AR-ir cells coexpressing EGFP	14±3% (n = 3)	21±3% (n = 3)	No

**Figure 7:**
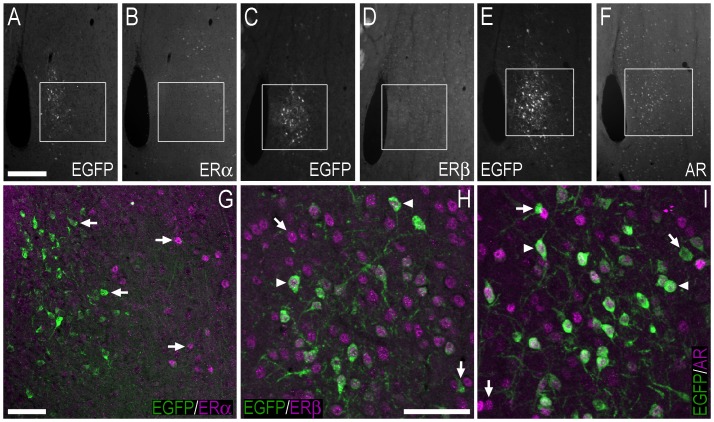
Double-immunofluorescence photo and confocal micrographs showing the distribution of EGFP−, ERα−, ERβ-, and AR-LI in the lateral septal nucleus. (**A, B**) Single-channel photomicrographs of double-fluorescence immunohistochemistry, showing EGFP− (A) and ERα-LI (B) (Bregma, +0.74 mm). (**C, D**) Single-channel photomicrographs of double-fluorescence immunohistochemistry, showing EGFP− (C) and ERβ-LI (D) (Bregma, +0.62 mm). (**E, F**) Single-channel photomicrographs of double-fluorescence immunohistochemistry, showing EGFP− (E) and AR-LI (F) (Bregma, +0.62 mm). (**G–I**) Confocal micrographs of double-immunofluorescence histochemistry displaying (G) EGFP− (green) and ERα-LI (magenta), (H) EGFP− (green), and ERβ-LI (magenta), and (I) EGFP− (green) and AR-LI (magenta). Confocal micrographs in (G), (H) and (I) are magnified views of the white squares shown in (A, B), (C, D) and (E, F), respectively. In G–I, arrows point to single labelled cell bodies, and arrowheads point to double-labelled cell bodies. Scale bars: A = 200 µm, applies A–F; G = 50 µm; H = 50 µm, applies H, I.

Cell bodies immunoreactive for ERα, ERβ, and the AR were also distributed in the LS, BST and OT. In the LS, coexpression between EGFP+ and ERα+ cell bodies was minimal, with most EGFP+ cells located more laterally than those expressing ERα (Fig. 7A, 7B, 7G). There were, however, occasional cell bodies in the LS that coexpressed EGFP− and ERα-LI. The LS contained a greater number of ERβ- or AR-ir cell bodies compared to those that expressed EGFP-LI (Fig. 7C, 7D, 7H; 7E, 7F, 7I), with most of the EGFP+ cell bodies also expressing ERβ-LI or AR-LI (Fig. 7H, 7I).

**Figure 8:**
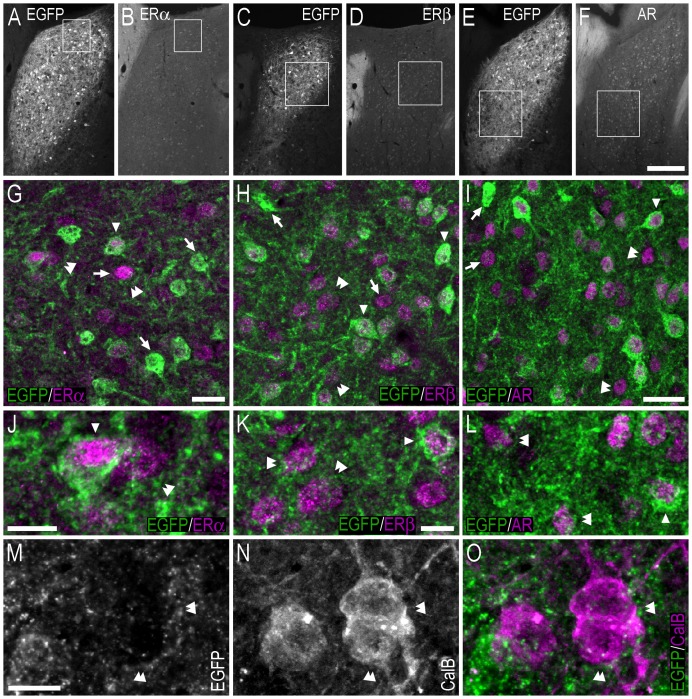
Double-immunofluorescence photo and confocal micrographs showing the distribution of EGFP-, ERα−, ERβ-, AR- and calbindin-LI in the bed nucleus of the stria terminalis. (**A, B**) Single-channel photomicrographs of double-fluorescence immunohistochemistry, showing EGFP− (A) and ERα-LI (B) (Bregma, −0.22 mm). (**C, D**) Single-channel photomicrographs of double-fluorescence immunohistochemistry, showing EGFP− (C) and ERβ-LI (D) (Bregma, −0.34 mm). (**E, F**) Single-channel photomicrographs of double-fluorescence immunohistochemistry, showing EGFP− (E) and AR-LI (F) (Bregma, −0.10 mm). (**G–I**) Confocal micrographs of double-immunofluorescence histochemistry displaying (G) EGFP− (green) and ERα-LI (magenta), (H) EGFP− (green), and ERβ-LI (magenta), and (I) EGFP− (green) and AR-LI (magenta). Confocal micrographs in (G), (H) and (I) are magnified views of the white squares shown in (A, B), (C, D) and (E, F), respectively. (**J–L**) Confocal micrographs of double-immunofluorescence histochemistry displaying (J) EGFP− (green) and ERα-LI (magenta), (K) EGFP− (green), and ERβ-LI (magenta), and (L) EGFP− (green) and AR-LI (magenta). The confocal micrographs in (J, K, L) are magnified views of the immunohistochemistry presented in (G, H, I), respectively. (**M–O**) Single-channel confocal micrographs of double-fluorescence immunohistochemistry (O), displaying EGFP− (M), and calbindin-LI (N). In G–L and M–O, arrows point to single labelled cell bodies, arrowheads point to double-labelled cell bodies, and double arrowheads point to apparent contacts or close anatomical association between EGFP-ir nerve fibres and single-labelled ERα− (G, J), ERβ- (H, K), AR- (I, L) or calbindin-ir (O) cell bodies. Scale bars: F = 200 µm, applies A–F; G = 20 µm; I = 25 µm, applies H, I; J = 10 µm; K = 10 µm, applies K, L; M = 10 µm, applies M–O.

In both female and male mice, the estimated number of ERα−, ERβ- or AR-ir cell bodies in the BST was higher than the number of EGFP+ cells (Table 2). While many of the EGFP+ cells co-expressed ERα−, ERβ- or AR-LI, EGFP+-only cell bodies were also found (Fig. 8A, 8B, 8G; 8C, 8D, 8H; 8E, 8F, 8I). The BST of female mice contained 36% fewer EGFP/ERα coexpressing cell bodies than male mice, 60% fewer EGFP/ERβ coexpressing cell bodies and 69% fewer EGFP/AR coexpressing cell bodies (Fig. 6B; Table 2). The proportion of EGPF+ cell bodies in the BST that coexpressed ERα-LI was greater in female than male mice, while the proportion of EGFP+ cell bodies in the BST that coexpressed ERβ-LI or AR-LI was similar in female and male mice (Fig. 6C; Table 2). The proportion of ERα-ir cell bodies in the BST that coexpressed EGFP was similar in female and male mice, as was the proportion of AR-ir cell bodies in the BST that were EGFP+ (Fig. 6D; Table 2). However, the proportion of ERβ-ir cell bodies in the BST that coexpressed EGPF was greater in male mice (Fig. 6D; Table 2).

Confocal analysis indicated that EGFP-ir fibres and terminal-like structures were often distributed adjacent to single-labelled ERα−, ERβ- or AR-ir cell bodies in the BST (Fig. 8G–I, 8J–L). When the positioning of these structures was further examined, with the addition of immunohistochemistry against calbindin (which is distributed throughout the cytoplasm and thus outlines the boundaries of the cell), it became apparent that EGFP-ir fibre/terminal-like structures were adjacent to the cell bodies in a way that suggested that they were in contact (Fig. 8M–O).

**Figure 9:**
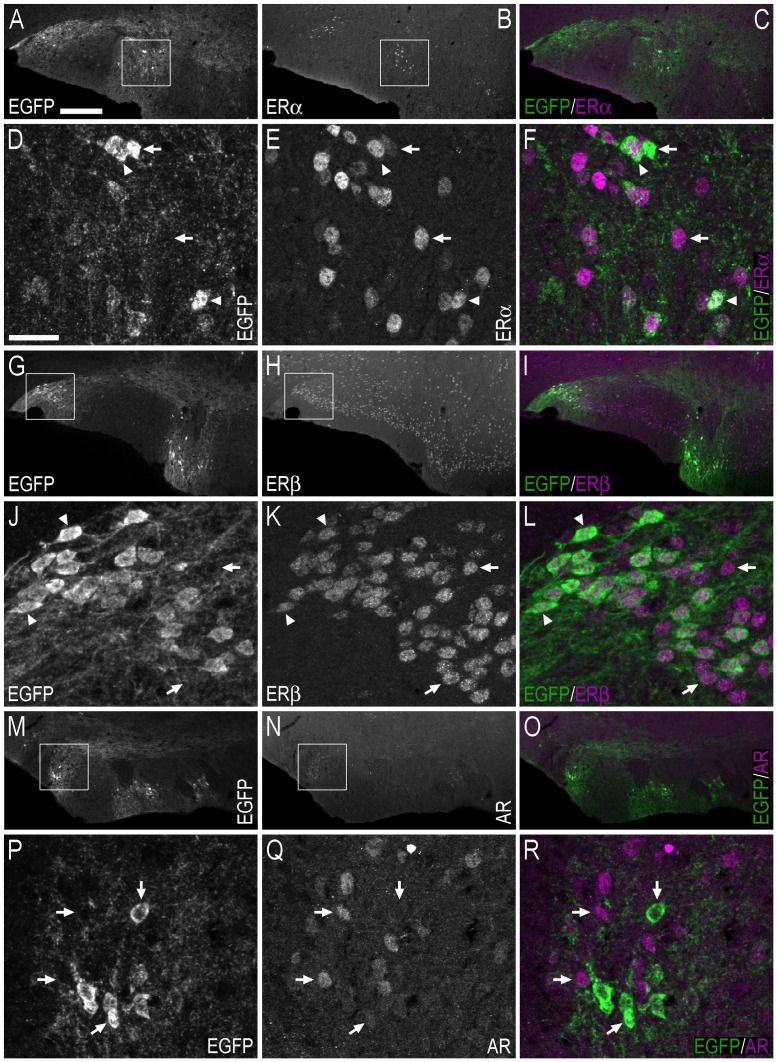
Double-immunofluorescence photo and confocal micrographs showing the distribution of EGFP-, ERα−, ERβ-, and AR-LI at the level of the olfactory tubercle. (**A–C**) Single-channel photomicrographs of double-fluorescence immunohistochemistry (C), showing EGFP− (A) and ERα-LI (B) (Bregma, +0.38 mm). (**D–F**) Single-channel confocal micrographs of double-immunofluorescence (F), displaying EGFP− (D), and ERα-LI (E). The confocal micrographs in (D–F) are magnified views from the white squares shown in (A) and (B). (**G–I**) Single-channel photomicrographs of double-fluorescence immunohistochemistry (I), showing EGFP− (G) and ERβ-LI (H) (Bregma, +0.38 mm). (**J–L**) Single-channel confocal micrographs of double-immunofluorescence (L), displaying EGFP− (J), and ERβ-LI (K). Confocal micrographs in (J–L) are magnified views of the white squares shown in (G) and (H). (**M–O**) Single-channel photomicrographs of double-fluorescence immunohistochemistry (O), showing EGFP− (M) and AR-LI (N) (Bregma, +1.42 mm). (**P–R**) Single-channel confocal micrographs of double-immunofluorescence (R), displaying EGFP− (P), and AR-LI (Q). Confocal micrographs in (P–R) are magnified views of the white squares shown in (M) and (N). In D–F, J–L and P–R, arrows point to single labelled cell bodies, and arrowheads point to double-labelled cell bodies. Scale bars: A = 200 µm, applies A–C, G–I, M–O; D = 25 µm, applies D–F, J–L, P–R.

In the Tu, many EGFP+ cell bodies coexpressed ERα-LI, although EGFP+-only cells were also present (Fig. 9A–F). Incidences of EGFP+ cell bodies that coexpressed ERβ-LI were also observed (Fig. 9G–L), while EGFP+ cell bodies that coexpressed AR-LI were not identified in the Tu (Fig. 9M–R).

#### Cerebral Cortex

In the cerebral cortex, EGFP+ cell bodies were infrequently observed in the cingulate cortex and the secondary motor cortex.

#### Hippocampal Formation

Occasional, sparsely distributed, EGFP+ cell bodies and EGFP-ir fibres were observed in the stratum radiatum (Rad) of the hippocampal formation (Fig. 4F, 4G), which did not coexpress ERα−, ERβ-, or AR-LI.

#### Amygdaloid complex

A high density of strongly EGFP-ir nerve fibres was found throughout the MePD and MePV of male mice (Figs. 3L, 4H). In female mice, a lower density of comparatively weaker EGFP-ir fibres was present (Fig. 3K). There was moderate EGFP-LI in nerve fibres in other regions of the amygdaloid complex of male and female mice (Table 1), including the anterior cortical amygdaloid nucleus (ACo) (Fig. 4I), posteromedial cortical amygdaloid nucleus (PMCo) (Fig. 4K), and the medial amygdaloid nucleus – anterior dorsal (MeAD), anteroventral (MeAV) and anterior parts (MeA) (Fig. 4I). Immunoreactivity was weaker in nerve fibres within the amygdalohippocampal area (AHi) (Fig. 4K), anterior amygdaloid area – dorsal (AAD) and ventral parts (AAV) (Fig. 5B), central amygdaloid nucleus – medial division (CeM) (Fig. 4L), bed nucleus of the stria terminalis – intraamygdaloid division (BSTIA) (Fig. 4L), and posterolateral cortical amygdaloid nucleus (PLCo) (Fig. 4K).

**Figure 10:**
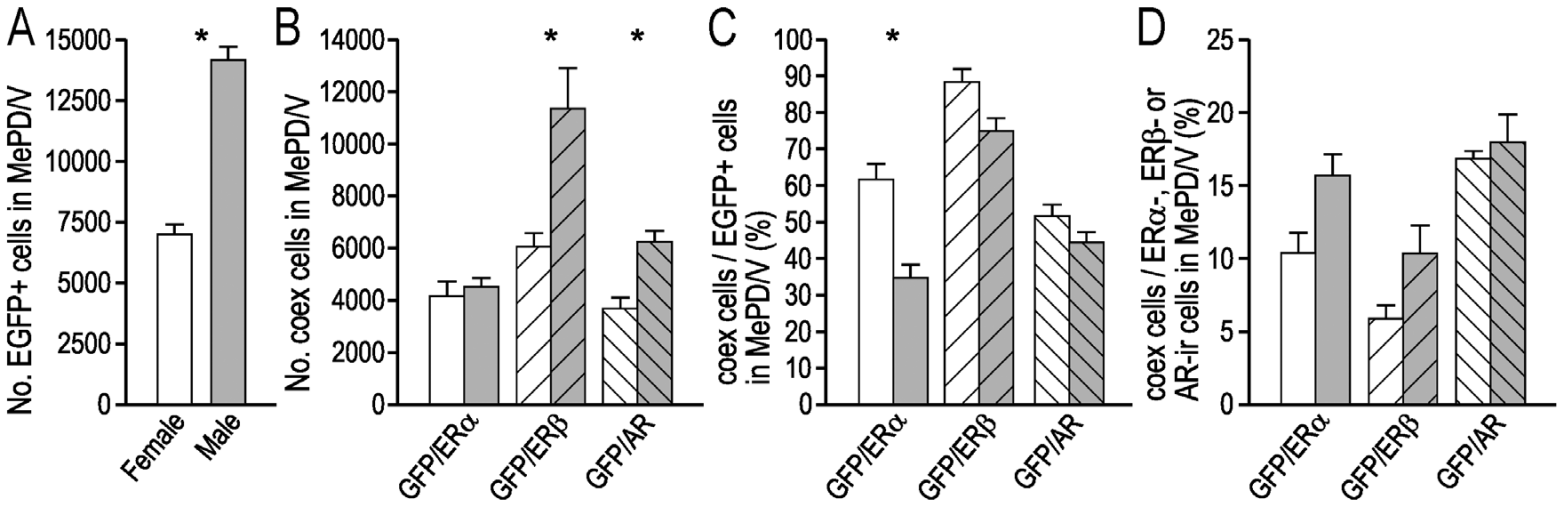
Quantification of EGFP+, ERα−, ERβ- and AR-ir cell bodies in the female and male MePD/MePV. (**A**) Estimated number of EGFP+ cell bodies in the female and male MePD/MePV (n = 5 per group). (**B**) Estimated number of EGFP+ cell bodies in the MePD/MePV that coexpressed ERα, ERβ or AR-LI (n = 3 per group). (**C**) The number of EGFP+ cell bodies in the MePD/MePV coexpressing ERα, ERβ or AR-LI, expressed as a percentage of the estimated number of EGFP+ cell bodies in the MePD/MePV (n = 3 per group). (**D**) The number of EGFP+ cell bodies in the MePD/MePV coexpressing ERα, ERβ or AR-LI, expressed as a percentage of the estimated number of ERα, ERβ or AR-ir cell bodies in the MePD/MePV (n = 3 per group). In A–D, white bars = female mice, grey bars = male mice, * corresponds to *P*<0.05 (student *t*-test).

The number of EGFP+ cell bodies in the MePD and MePV of the female mouse was 51% lower than the estimated number in male mice (Figs. 3K, 3L, 10A, *P*≤0.05, Table 3). Low densities of EGFP+ cell bodies were also distributed in the ACo (Fig. 4I) and PMCo (Fig. 4K) of both male and female mice, with infrequent EGFP+ cell bodies dispersed through the MeA (Fig. 4I), CeM (Fig. 4L), BSTIA (Fig. 4L), and PLCo (Fig. 4K).

**Table 3:** Estimated number of EGFP+, ERα-ir, ERβ-ir and AR-ir cell bodies in the MePD/V (mean ± SEM).

MePD/V			
	Female	Male	*P*≤0.05
No. EGFP+ cells	6984±407 (n = 5)	14148±550 (n = 5)	Yes
No. ERα-ir cells	40200±2257 (n = 3)	29100±2979 (n = 3)	Yes
No. ERβ-ir cells	105500±8147 (n = 3)	111650±5572 (n = 3)	No
No. AR-ir cells	21760±1960 (n = 3)	35610±4442 (n = 3)	Yes
No. EGFP+/ERα-ir coexpressing cells	4150±577 (n = 3)	4500±346 (n = 3)	No
No. EGFP+/ERβ-ir coexpressing cells	6050±522 (n = 3)	11350±1540 (n = 3)	Yes
No. EGFP+/AR-ir coexpressing cells	3680±419 (n = 3)	6240±416 (n = 3)	Yes
Proportion EGFP+ cells coexpressing ERα-ir	62±4% (n = 3)	35±4%% (n = 3)	Yes
Proportion EGFP+ cells coexpressing ERβ-ir	88±4% (n = 3)	75±4% (n = 3)	No
Proportion EGFP+ cells coexpressing AR-ir	52±3% (n = 3)	44±3% (n = 3)	No
Proportion ERα-ir cells coexpressing EGFP	10±1% (n = 3)	16±1% (n = 3)	No
Proportion ERβ-ir cells coexpressing EGFP	6±1% (n = 3)	10±2% (n = 3)	No
Proportion AR-ir cells coexpressing EGFP	17±1% (n = 3)	18±2% (n = 3)	No

Cell bodies immunoreactive for ERα, ERβ, and the AR were also distributed through the MePD and MePV, and their number was greater than the number of EGFP+ cells (Table 3). In these regions of the amygdaloid complex, female mice contained 47% fewer EGFP/ERβ coexpressing cell bodies than male mice and 41% fewer EGFP/AR coexpressing cell bodies, while the number of EGFP/ERα coexpressing cell bodies was similar in female and male mice (Fig. 10B; Table 3). The proportion of EGPF+ cell bodies in the MePD and MePV that coexpressed ERα-LI was greater in female than male mice, while the proportion of EGPF+ cell bodies in the BST that coexpressed ERβ-LI or AR-LI was similar in female and male mice (Fig. 10C; Table 3). The proportion of ERα-ir cell bodies in the MePD and MePV that coexpressed EGFP was similar in female and male mice, as was the proportion of ERβ-ir and AR-ir cell bodies that coexpressed EGFP (Fig. 10D; Table 3).

**Figure 11:**
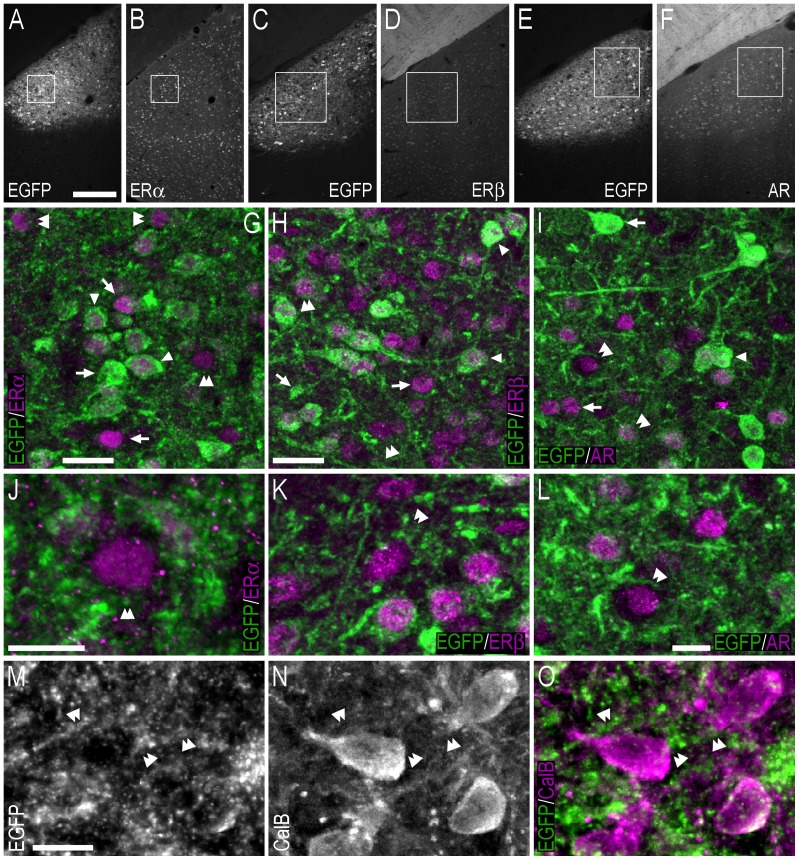
Double-immunofluorescence photo and confocal micrographs showing the distribution of EGFP−, ERα−, ERβ- AR- and calbindin-LI in the medial amygdaloid nucleus – posterodorsal (MePD) part. (**A, B**) Single-channel photomicrographs of double-fluorescence immunohistochemistry, showing EGFP− (A) and ERα-LI (B) (Bregma, −2.06 mm). (**C, D**) Single-channel photomicrographs of double-fluorescence immunohistochemistry, showing EGFP− (C) and ERβ-LI (D) (Bregma, −2.06 mm). (**E, F**) Single-channel photomicrographs of double-fluorescence immunohistochemistry, showing EGFP− (E) and AR-LI (F) (Bregma, −2.06 mm). (**G–I**) Confocal micrographs of double-immunofluorescence histochemistry displaying (G) EGFP− (green) and ERα-LI (magenta), (H) EGFP− (green), and ERβ-LI (magenta), and (I) EGFP− (green) and AR-LI (magenta). Confocal micrographs in (G), (H) and (I) are magnified views of the white squares shown in (A, B), (C, D) and (E, F), respectively. (**J–L**) Confocal micrographs of double-immunofluorescence histochemistry displaying (J) EGFP− (green) and ERα-LI (magenta), (K) EGFP− (green), and ERβ-LI (magenta), and (L) EGFP− (green) and AR-LI (magenta). The confocal micrographs in (J, K, L) are magnified views of the immunohistochemistry presented in (G, H, I), respectively. (**M–O**) Single-channel confocal micrographs of double-fluorescence immunohistochemistry (O), displaying EGFP− (M), and calbindin-LI (N). In G–L and M–O, arrows point to single labelled cell bodies, arrowheads point to double-labelled cell bodies, and double arrowheads point to apparent contacts or close anatomical association between EGFP-ir nerve fibres and single-labelled ERα− (G, J), ERβ- (H, K), AR-(I, L) or calbindin-ir (O) cell bodies. Scale bars: A = 200 µm, applies A–F; G = 20 µm; H = 25 µm, applies H, I; J = 10 µm; L = 10 µm, applies K, L; M = 10 µm, applies M–O.

As was the case in the BST, confocal analysis showed that EGFP-ir fibres and terminal-like structures were often adjacent to single-labelled ERα−, ERβ- or AR-ir cell bodies in the MePD and MePV (Fig. 11G–I, 11J–L). As described previously, apparent contacts between EGFP-ir fibres and adjacent lying cell bodies were observed following immunohistochemistry against calbindin (Fig. 11M–O).

### Diencephalon

#### Hypothalamus

**Figure 12:**
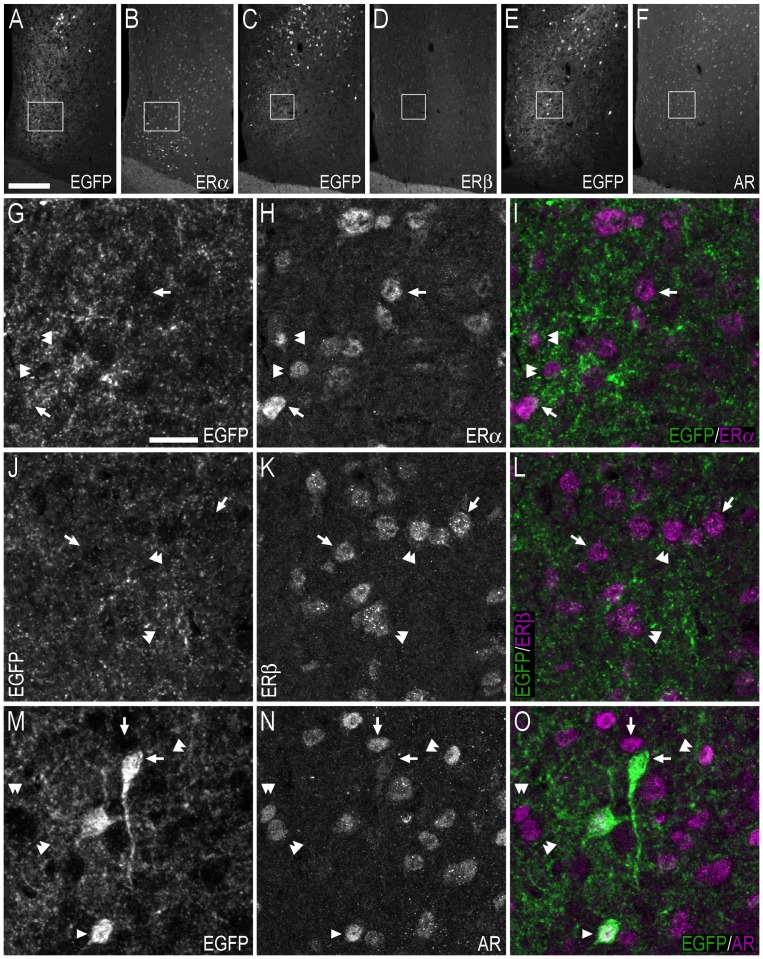
Double-immunofluorescence photo and confocal micrographs showing the distribution of EGFP-, ERα−, ERβ-, and AR-LI at the level of the medial preoptic nucleus/area. (**A, B**) Single-channel photomicrographs of double-fluorescence immunohistochemistry, showing EGFP− (A) and ERα-LI (B) (Bregma, −0.22 mm). (**C, D**) Single-channel photomicrographs of double-fluorescence immunohistochemistry, showing EGFP− (C) and ERβ-LI (D) (Bregma, −0.34 mm). (**E, F**) Single-channel photomicrographs of double-fluorescence immunohistochemistry, showing EGFP− (E) and AR-LI (F) (Bregma, −0.10 mm). (**G–I**) Single-channel confocal micrographs of double-immunofluorescence (I), displaying EGFP− (G), and ERα-LI (H). The confocal micrographs in (G–I) are magnified views from the white squares shown in (A) and (B). (**J–L**) Single-channel confocal micrographs of double-immunofluorescence (L), displaying EGFP− (J), and ERβ-LI (K). Confocal micrographs in (J–L) are magnified views of the white squares shown in (C) and (D). (M–O) Single-channel confocal micrographs of double-immunofluorescence (O), displaying EGFP− (M), and AR-LI (N). Confocal micrographs in (**M–O**) are magnified views of the white squares shown in (E) and (F). In G–O, arrows point to single labelled cell bodies, arrowheads point to double-labelled cell bodies, and double arrowheads point to apparent contacts or close anatomical association between EGFP-ir nerve fibres and single-labelled ERα− (I), ERβ- (L) or AR-ir (O) cell bodies. Scale bars: A = 200 µm, applies A–F; G = 20 µm, applies G–O.

There was a very high density of strongly EGFP-ir nerve fibres in the medial preoptic nucleus (MPO) of the male hypothalamus (Fig. 3D), with a high density of fibres in the medial preoptic area (MPA) (Figs. 3D, 12E), medial tuberal nucleus (MTu) (Fig. 3J) and terete hypothalamic nucleus (Te) (Fig. 5F), and a moderate density of fibres in the ventromedial preoptic nucleus (VMPO) (Fig. 5A). A lower density of EGFP-ir fibres was observed in these regions of the female mouse (Fig. 3C c.f. 3D, 3I c.f. 3J, Table 1). The density of EGFP-ir fibres was similar in other regions of the male and female hypothalamus (Table 1). EGFP-ir fibre density was: high in the supraoptic nucleus (SO) (Figs. 4I, 5B) and premammillary nucleus – ventral part (PMV) (Fig. 5C); moderate in the anterior hypothalamic area (AH) (Fig. 5D, 5H) and lateroanterior hypothalamic nucleus (LA) (Fig. 5D), dorsomedial hypothalamic nucleus (DM) (Fig. 5E, 5G) and perifornical area (PeF) (Fig. 5E, 5F); and low in regions including the arcuate hypothalamic nucleus (Arc) (Fig. 5C, 5G), lateral hypothalamic area (LH) (Fig. 5B, 5F), paraventricular hypothalamic nucleus (Pa) (Fig. 5H), premammillary nucleus – dorsal part (PMD) (Fig. 5C), and the tuber cinereum area (TC) (Fig. 5I).

**Figure 13:**
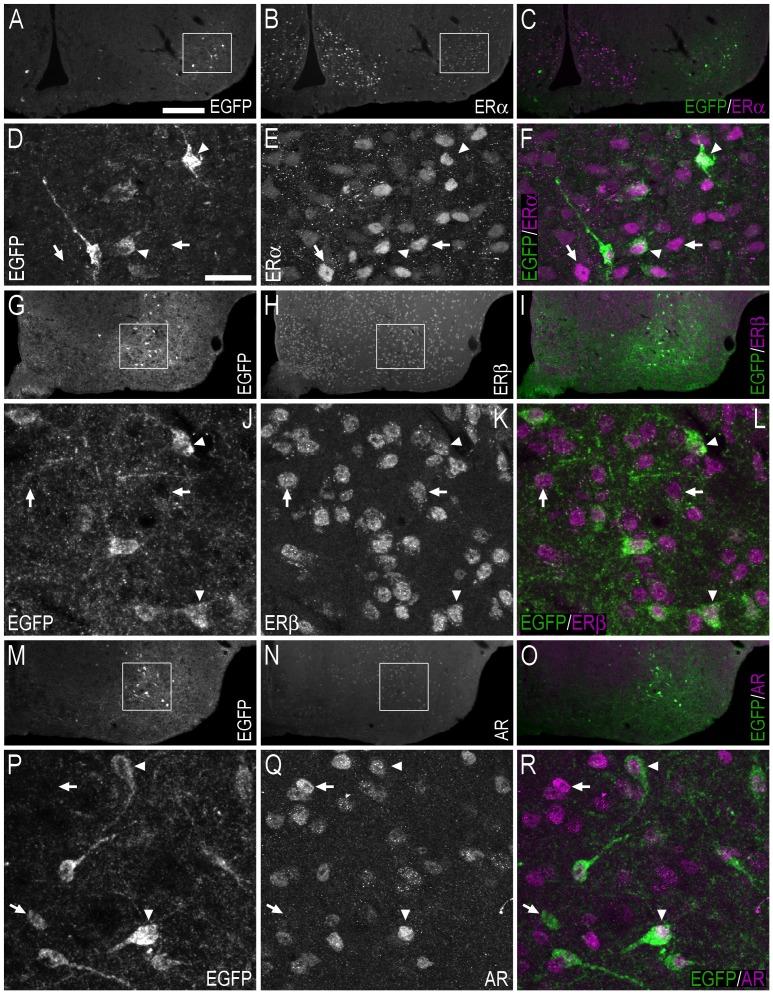
Double-immunofluorescence photo and confocal micrographs showing the distribution of EGFP−, ERα−, ERβ-, and AR-LI in the hypothalamus. (**A–C**) Single-channel photomicrographs of double-fluorescence immunohistochemistry (C), showing EGFP− (A) and ERα-LI (B) (Bregma, −1.94 mm). (**D–F**) Single-channel confocal micrographs of double-immunofluorescence (F), displaying EGFP− (D), and ERα-LI (E). The confocal micrographs in (D–F) are magnified views from the white squares shown in (A) and (B). (**G–I**) Single-channel photomicrographs of double-fluorescence immunohistochemistry (I), showing EGFP− (G) and ERβ-LI (H) (Bregma, −1.94 mm). (**J–L**) Single-channel confocal micrographs of double-immunofluorescence (L), displaying EGFP− (J), and ERβ-LI (K). Confocal micrographs in (J–L) are magnified views of the white squares shown in (G) and (H). (**M–O**) Single-channel photomicrographs of double-fluorescence immunohistochemistry (O), showing EGFP− (M) and AR-LI (N) (Bregma, −1.46 mm). (**P–R**) Single-channel confocal micrographs of double-immunofluorescence (R), displaying EGFP− (P), and AR-LI (Q). Confocal micrographs in (P–R) are magnified views of the white squares shown in (M) and (N). In D–F, J–L, P–R, arrows point to single labelled cell bodies, and arrowheads point to double-labelled cell bodies. Scale bars: A = 200 µm, applies A–C, G–I, M–O; D = 25 µm, applies D–F, J–L, P–R.

Hypothalamic nuclei harbouring EGFP+ cell bodies included the MPO (Fig. 3C, 3D) and MPA (Figs. 12E, 12M, 5A), where higher numbers of EGFP+ cell bodies were present in male mice (Table 1). The density of EGFP+ cell bodies appeared similar in the DM of male and female mice (Fig. 5E), while infrequent, sparsely distributed, EGFP+ cell bodies were found in regions including the AH (Fig. 5D), Arc (Fig. 5C, 5G), LH (Fig. 5F), Pa, PeF (Fig. 5E, 5F), TC, VMPO (Fig. 5A), and the MTu/Te (Figs. 3I, 3J, 13A, 13G, 13M).

**Figure 14:**
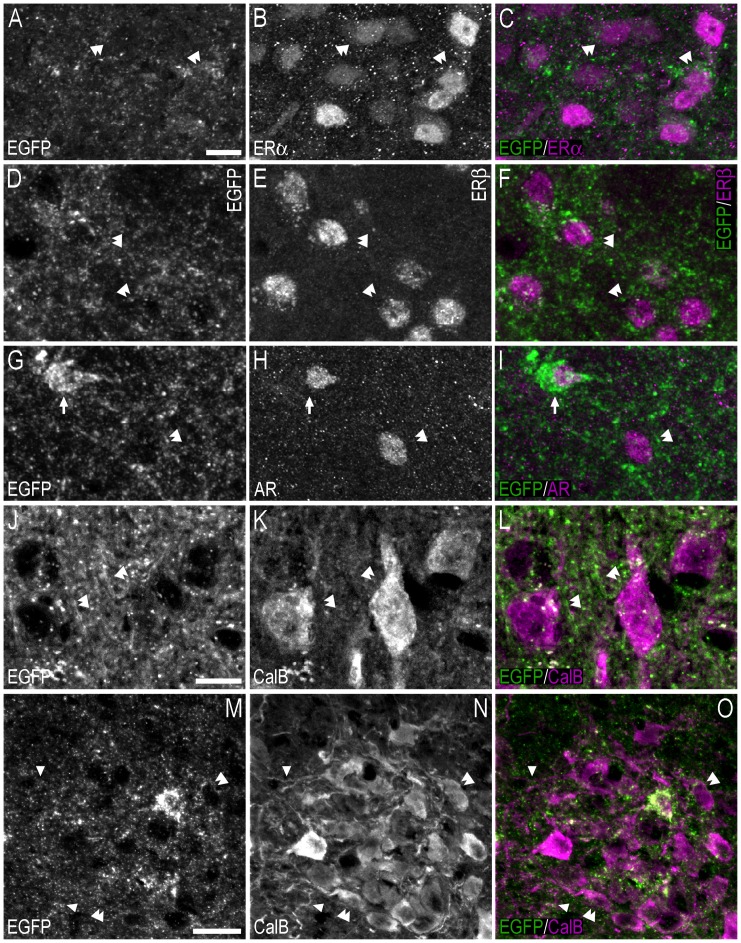
Double-immunofluorescence confocal micrographs showing apparent contacts between EGFP+ fibres/terminal-like structures, and ERα−, ERβ-, AR- or calbindin-ir cell bodies/fibres in the hypothalamus. (**A–C**) Single-channel confocal micrographs of double-fluorescence immunohistochemistry (C), displaying EGFP− (A), and ERα-LI (B). The confocal micrographs in (A–C) are magnified views of the immunohistochemistry presented in Fig. 9A–F. (**D–F**) Single-channel confocal micrographs of double-fluorescence immunohistochemistry (F), displaying EGFP− (D), and ERβ-LI (E). Confocal micrographs in (D–F) are magnified views of Fig. 9G-L. (**G–I**) Single-channel confocal micrographs of double-fluorescence immunohistochemistry (I), displaying EGFP− (G), and AR-LI (H). Confocal micrographs in (G–I) are magnified views of Fig. 9M-R. (**J–L**) Single-channel confocal micrographs of double-fluorescence immunohistochemistry (L), displaying EGFP− (J), and calbindin-LI (K). (**M–O**) Single-channel confocal micrographs of double-fluorescence immunohistochemistry (O), displaying EGFP− (M), and calbindin-LI (N). In A–O, arrows point to double-labelled cell bodies, arrowheads point to apparent contacts or close anatomical association between EGFP-ir nerve fibres and calbindin-ir fibres (O), and double arrowheads point to apparent contacts or close anatomical association between EGFP-ir nerve fibres and single-labelled ERα− (C), ERβ- (F), AR-ir (I) or calbindin-ir (L, O) cell bodies. Scale bars: A = 10 µm, applies A–I; J = 10 µm, applies J–L; M = 20 µm, applies M–O.

Cell bodies immunoreactive for ERα and the AR were found in the hypothalamic nuclei containing EGFP+ cells, including the MPO, MPA, LH, PeF, MTu and Te (Figs. 12 and 13). ERβ-ir cell bodies were also present in the MPO and MPA, as well as the arcuate nucleus, LH and adjacent MTu, PeF and Te. In double-labelling experiments the few, sparsely distributed, EGFP+ cell bodies in the MPO did not coexpress ERα-LI (Fig. 12A, 12B, 12G–I). However, while some EGFP+ cells in the MPO coexpressed ERβ-LI and AR-LI, EGFP+-only cell bodies were also identified (Fig. 12C, 12D, 12J–L; 128E, 12F, 12M–O). In the MTu/Te/PeF region, all identified EGFP+ cell bodies coexpressed ERα-LI (Fig. 13A–F), ERβ-LI (Fig. 13G–L) or AR-LI (Fig. 13M–R). Confocal analysis showed that EGFP-ir fibres and terminal-like structures in the MPO, MPA, LH, and the MTu and adjacent nuclei, were often in close anatomical association with, and apparently contacting, single-labelled ERα−, ERβ- or AR-ir cell bodies (Figs. 12G–I, 12J–L, 12M–O; 13D–F, 13J–L, 13P–R; 14A–C; 14D–F, 14G–I) and cell bodies containing calbindin-LI (Fig. 14J–O).

#### Thalamus

In the thalamus, some sparsely distributed EGFP+ cell bodies were found in the A13 region of zona incerta (ZI), in both male and female mice (Fig. 4M). A low density of weakly EGFP-ir nerve fibres was also observed in the ZI.

## Discussion

As the last step in oestrogen synthesis is aromatisation and as most oestrogen effects are mediated through oestrogen receptors, attempts have been made to compare the distributions of aromatase and oestrogen receptors in the brain [29]–[32]. However, a description of the distribution of aromatase in the rodent brain, and its coexpression with oestrogen and androgen receptors in individual cells, has been hindered by the lack of sensitive and specific antibodies. This problem has been circumvented by tagging aromatase expression to reporter gene expression following the physiological activation of the *Cyp19A1* gene.

In the transgenic mouse model developed previously by Wu et al. [33], the nuclear LacZ reporting gene was integrated into the aromatase locus and the reporter protein is nuclear β-galactosidase, which identifies aromatase positive cell bodies but not the neurites extending from the soma. Since aromatase is a endoplasmic reticulum protein [34] and the endoplasmic reticulum has been reported to be present in synapses [35], we have adopted a different approach – to tag aromatase expression by a EGFP, a cytoplasmic protein, so that neurites extending from aromatase expressing neurones can be identified. This is made possible by inserting the EGFP cDNA before the ATG start codon of the aromatase coding region in the *Cyp19A1* gene. EGFP cDNA is expressed (i.e. transcribed and translated) whenever the aromatase gene is expressed. However, the resulting protein molecule of EGFP is not linked to the aromatase protein molecule, as indicted by the small molecular weight (∼32.7 kDa) of the expressed EGFP band on the Western blot analyses. Aromatase is an integral membrane protein of the endoplasmic reticulum, anchored to the membrane by an amino terminal transmembrane domain, whereas EGFP is a cytoplasmic protein. Although it is **not** our intention to describe the subcellular location of aromatase, EGFP is a good marker for neurites extending from aromatase positive neurones as it is present throughout the cytoplasm of aromatase-positive neurones. This logic is strengthened by the fact that EGFP fibres could not derive from neurones not expressing aromatase. A second advantage of not covalently linking EGFP to aromatase protein is to not alter aromatase activity.

We have identified cells expressing aromatase by EGFP immunoreactivity. Immunohistochemistry for EGFP was performed using the TSA^+^ technique [36], a highly sensitive modification of the original indirect technique developed by Coons and collaborators [37]. Antibodies to the ERα, ERβ and AR have also been validated, with Western blotting revealing a single band of predicted size, and staining patterns in agreement with previous immunohistochemical and *in situ* hybridization studies in mouse and rat [38]–[43]. Thus, the ERα, ERβ and AR antisera do not seem to be associated with the type of unspecificity described for antibodies to, for example, many GPCRs [44].

EGFP-LI was completely absent from brain tissue of wild-type mice in which transcription of EGFP was not induced by physiological activation of the *Cyp19A1* gene. Further support that this approach provides a valid representation of aromatase protein comes from the concordance between the distribution of EGFP-LI in the brain of this mouse and earlier *in situ* hybridization and aromatase activity studies performed in mouse, rat and monkey [2], [6]–[10], [18], [19], [21]. Specifically, in both sexes, aromatase expression was highest in cells and fibres of the bed nucleus of the stria terminalis, olfactory tubercle, and the posterodorsal and posteroventral parts of the medial amygdaloid nucleus. Nevertheless, differences with previous descriptions of the distribution of aromatase in rats were found: EGFP+ cell bodies in the hippocampal formation were uncommon and sparsely distributed (c.f. [2], [18]); infrequent EGFP+ cell bodies were found in the central amygdaloid nucleus (c.f. [21]); and EGFP-LI was not found in the cerebellum (c.f. [11]). These discrepancies may represent species differences. It is also possible that aromatase (or EGFP) protein is expressed in these regions but at a level that is below detection by our immunohistochemical approach. Note that a truncated form of aromatase has previously been detected in the rat cerebral cortex [45], [46], where little or no aromatase activity has been reported [19]. The location of the EGFP promoter used in this study would prevent the identification of this truncated form of aromatase, which has not been identified in human or mouse.

Our data, for the most part, corresponds well with the distribution of aromatase obtained from *in situ* hybridization data in the GENSAT atlas [27] and the Allen Brain Atlas [28]. For example, EGFP+ cell bodies and those expressing the *Cyp19A1* transcript were observed in the ventral striatum, BST and the medial amygdaloid nucleus. On the other hand, EGFP+ cell bodies were found in the lateral septal nucleus, but no *Cyp19A1* transcript was reported in this nucleus. We contend that EGFP-LI allows a more precise description of the specific nuclei in which cell bodies expressing aromatase are distributed, and thus expands on descriptions provided by GENSAT and the Allen Brain Atlas. However, a direct comparison is difficult because distribution of the *Cyp19A1* transcript in these atlases is obtained from sagittal sections, whereas our results were derived from examination of sections cut in the coronal plane.

While the expression of EGFP-LI within the various cell bodies of the hypothalamus reflected previous *in situ* hybridization [13], [47] and aromatase activity findings [6], [19], there was frequently a high density of EGFP-LI outside cell bodies, whose distribution corresponded to fibres or terminals. We suspect that these EGFP-ir fibres/terminals may reflect fibre projections originating from the BST and medial amygdala [48] to the various hypothalamic nuclei, in particular, e.g. the medial preoptic nucleus/area, and the premammillary, medial tuberal and supraoptic nuclei, which contained the highest density of EGFP-ir fibres. Stronger EGFP-LI detected in the male hypothalamus may infer an increase in arbourisation of projections from distant aromatase positive neurones, such as the medial amygdaloid nucleus.

EGFP-LI and EGFP+ cell bodies were significantly higher in the BST and the medial amygdaloid nucleus of male mice than in the corresponding nuclei in female mice, and is in keeping with previous findings in a transgenic mouse with a LacZ reporter cassette inserted into the 3′UTR of the *Cyp19A1* gene [33]. Thus, it appears that there are more aromatase-expressing cells in these regions of the male mouse brain. Previously, differences in the number of cell bodies in the medial amygdaloid nucleus (and BST) of male and females was explained by sex-specific apoptosis, because more TUNEL-positive cells were found in females between P1 and P10 than in males of the same age [33], [49]. To summarise our data, adult female mice have fewer EGFP+ cell bodies in the BST that coexpress ERα, ERβ or the AR, and fewer EGFP+ cell bodies in the MePD/MePV that coexpress ERβ or the AR than adult male mice. This suggests that cells in the BST and MePD/MePV of female mice at P1–P10 that undergo apoptosis may be aromatase-containing cell bodies that coexpress ERα, ERβ and/or the AR. EGFP/ERα coexpressing cell bodies in the MePD/MePV may be an exception because their numbers in adult female and male mice were similar.

Of the three steroid receptors examined, ERβ-LI was most commonly coexpressed in EGFP+ cell bodies in the BST and MePD/MePV of female and male adult mice. We propose two functional implications of this high incidence of ERβ coexpression. The first relates to neurone survival. As discussed above, cells in the BST and medial amygdaloid nucleus of P1–P10 female and male mice undergo apoptosis [33], [49]. Oestrogen signalling though the ERβ may regulate the number of neurones in the BST and medial amygdaloid nucleus by promoting survival and reducing apoptotic cell death at this age. Indeed, Hisasue et al. [50] demonstrated that ERβ specific agonist diarylpropionitrile (DPN) increased BST cell number although not volume in C57BL6 female mice. If so, we expect the proportion of EGFP+ cell bodies expressing ERβ-LI to be similar in the BST and MePD/MePV of adult and newborn mice. Whether this is the case should be clarified in future investigations as it had been reported that ERβKO but not ERαKO mice maintain a similar sexually dimorphic BST as wild-type mice [51]. The second implication that we propose is that oestrogens, signalling through ERβ, influence aromatase expression in neurones in the BST and MePD/MePV of the adult mouse, and can thus promote male behaviours [52]. In view of the close proximity of the aromatase or rather EGFP labelling and oestrogen receptor labelling observed, this may be through an autocrine mechanism, where oestrogens produced by aromatase-expressing cells act on ERβ expressed by these cells (∼88% and ∼75% of EGFP+ cells coexpress ERβ in the female and male MePD/MePV, respectively), or through paracrine signalling, where oestrogens released by non-ERβ/aromatase-producing cells act on aromatase/ERβ coexpressing cells (∼12% and ∼25% of EGFP+ cells were not found to express ERβ in the female and male MePD/MePV, respectively).

By discussing possible functional attributes of ERβ, we do not preclude roles for ERα or the AR in these events. In fact, Wu and colleagues [33] reported that in newborn mice of both sexes, 99% of β-galactosidase positive (aromatase) cells co-express ERα in the BST and medial amygdaloid nucleus. In adult mice however, our current study demonstrates that only ∼35% of EGFP+ cells coexpressed ERα in the male MePD/MePV (∼29% in male BST), while ∼62% of EGFP+ cells coexpressed ERα in the female (∼60% in female BST); both are significantly lower than reported previously in newborn mice [33]. Changes in these proportions, and the higher levels of coexpressing EGFP/ERα cell bodies in female mice in particular, indicate that signalling through ERα is also important in regulating cell survival, levels of aromatase and male behaviours [52], [53]. However, a single cell body can express more than one of the three (ERα, ERβ or the AR) receptors, thus adding complexity to this discussion. In fact, a single cell may express all three receptor subtypes and regions of the female and male postnatal brain expressing ERα and ERβ overlap [54]. Thus, to gain further insight on the precise roles for ERα, ERβ and the AR, further studies are required to compare the number, and proportion, of aromatase cells expressing ERα, ERβ and/or the AR in the newborn/postnatal and adult brain.

The distribution of aromatase positive neurones observed in our study is similar to that described in Wu et al. [33] and we proceeded to examine how this EGFP-positive fibre distribution pattern integrated with oestrogen and androgen receptors. As the EGFP is not fused to the aromatase protein, which is endoplasmic reticulum bound, there is the advantage that fibres of aromatase expressing cells can be identified. This provides clues to the location of terminals and fibres of aromatase expressing cells with respect to the cell somata that were immunoreactive for ERα, ERβ or the AR. Confocal microscopy revealed EGFP-ir structures, with a fibre/terminal-like appearance, lying in close proximity to cell bodies immunoreactive for ERα, ERβ or the AR. This observation supports the current view that oestrogens derived from neural aromatisation of testosterone have paracrine, intracrine or autocrine influences on ERα and ERβ [26], [55]–[57]. The coexpression of AR-LI and EGFP-LI (i.e. aromatase) in cell bodies supports previous reports that androgen regulates brain aromatase expression [14], [21], [47], [58].

As neural aromatisation of testosterone is thought to have organisation roles i.e. to masculinise and defeminise the brain [55], [56], future analyses of aromatase-EGFP expression in our transgenic mouse embryonic brains will address whether there is a sexually dimorphic expression pattern similar to the adult brains. The high levels of aromatase in the BST and the medial amygdaloid nucleus of adult male mice suggest that these nuclei are important in masculine behaviours, i.e. activational roles as supported by reports that male sexual behaviour is impaired in the male aromatase knockout (ArKO) mouse [59] but can be restored in adulthood by oestrogen replacement [60]. Furthermore, we can postulate that the sexual differences of aromatase, oestrogen receptors and androgen receptor described here in gonadally intact animals may be altered by circulating hormones. Future studies will be conducted to investigate the changes in the distribution during hormonal manipulation either by gonadectomy or hormone/agonist/antagonist administration.

There were moderate levels of EGFP expression in the caudomedial regions of the caudate putamen and the A13 region of the zona incerta of the thalamus. Aromatase expression has been previously described in the caudate and putamen and thalamus of the human brain in a positron emission tomography study using the radiolabelled aromatase inhibitor [N-methyl-(11)C]vorozole [61].

In summary, the widespread distribution of EGFP+ cell bodies and immunoreactive fibres suggests that aromatase expression is common in the mouse brain and is in close proximity to oestrogen receptors. This supports the long standing notion that locally synthesized oestrogens mediate biological effects by paracrine and autocrine activation of pre- and post-synaptic oestrogen α and β receptors, and androgen receptors, and also by projections to distant nuclei, particularly in the hypothalamus. The sex differences in aromatase expression in regions such as the amygdala and BST suggest that these nuclei may be important in producing differences in male and female behaviours.

## Materials and Methods

### Animals

All experimental procedures performed in this study conformed to the Australian National Health and Medical Research Council published code of practice, and were approved by the Animal Ethics Committee of The Florey Institute of Neuroscience and Mental Health (#09-024). The transgenic CYP19A1-EGFP BAC-mouse strain (Swiss 6X FVB/N) was obtained from the Mutant Mouse Regional Resource Centers at University of California, Davis, USA. An EGFP gene was inserted upstream of the ATG start codon of the *Cyp19A1* gene of the selected BAC clone [62]. The transgenic mouse line was back-crossed onto FVB/N background for >8 generations and maintained under specific pathogen-free (SPF) conditions on a 12 h day/night cycle, with water and soy-free food (Glen Forrest Stockfeeders, Glen Forrest, Western Australia, Australia) *ad libitum*. PCR genotyping was performed using primers:

EGFP-forward 5′ CCTACGGCGTGCAGTGCTTCAGC 3′

EGFP-reverse 5′ CGGCGAGCTGCACGCTGCCGTCCTC 3′.

Both male and female hemizygous transgenic animals have a gross anatomy, fertility, life span and hormonal profile similar to wild-type animals (data not shown). Eleven mature gonadally intact 16–20-weeks old hemizygous mice (6 male and 5 female), and six wild-type mice which do not have the EGFP transgene (3 male and 3 female), weighing between 35–40 g, were used. The weight of the transgenic mice was similar to wild-type littermates. In addition, the FVB/N mouse strain is not classified as obese, and these mice are less susceptible to obesity development than C57BL6 mice [63].

### Western blot analysis

#### Tissue preparation

All animals were deeply anaesthetised with pentobarbitone sodium (Lethabarb, Virbac, Milperra, NSW, Australia, 100 mg/kg i.p.) and killed by cervical dislocation. Brains were quickly removed and dissected, and samples snap frozen on dry ice and stored at −80°C until further processing.

#### Sample preparation

Tissue was homogenized by ultrasonication in TNE buffer (5 mM Tris-HCl, 0.5 mM EDTA, 0.1 M NaCl) also containing 0.32 M sucrose and protease inhibitors. Cell debris and nuclei were removed by centrifugation (800 g, 10 min at 4°C). Membrane and cytosol fractions were separated by centrifugation at 14,000 g for 30 min at 4°C. Membrane fractions were washed in TNE buffer containing protease inhibitors, and digested in TNE buffer supplemented with 1% Triton X-100 and protease inhibitors for 90 min. Undigested tissue was removed by centrifugation at 14,000 g for 30 min at 4°C. Protein concentrations were determined by Bradford's colorimetric method [64].

#### Gel electrophoresis and protein detection

**Figure 15:**
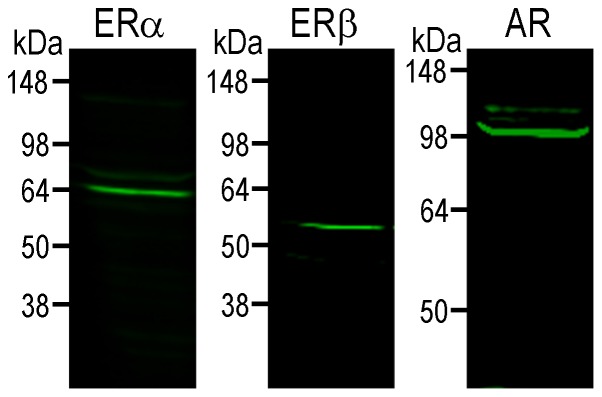
Western Blot of ERα, ERβ and AR using total brain protein extract. Single prominent bands at the expected sizes (ERα 66 kDa; ERβ 59 kDa; AR 99 kDa) were detected, confirming the specificities of the antibodies used.

Samples were diluted to a final protein concentration of 2 µg/µl in Laemmli buffer, and analyzed by SDS-PAGE on 10% resolving acrylamide gels. After transferring onto Odyssey nitrocellulose membranes (LI-COR), protein samples on membranes were blocked in 5% (w/v) non-fat milk in Tris-buffered saline solution containing 0.5% Tween-20 (TBST), pH 8.0, and exposed to primary antibodies [Rabbit anti-oestrogen receptor α (ERα) (C1355) polyclonal antibody, 1∶4,000, Millipore, Billerica, MA; Code No. 06-935; Lot No. NG1838275; Rabbit anti-oestrogen receptor β (ERβ) monoclonal antibody, 1∶4,000, Millipore; Code No. 05-824; Lot No. JBC1850147; Rabbit anti-androgen receptor (AR) PG-21 polyclonal antibody, 1∶4,000, Millipore; Code No. 06-680; Lot No. DAM1661059 (Table 4)] diluted in blocking solution, at 4°C overnight. Hybridized membranes then were washed 3 times in TBST for 5 mins, and probed with Odyssey secondary antibodies, IRDye 800CW Goat anti-Rabbit IgG and IRDye 680RD Goat anti-Mouse IgG (LI-COR) (1∶10,000). Immunoreactivity was visualised and analysed on the Odyssey Classic infrared imaging system (LI-COR; Fig. 15).

**Table 4:** Primary antisera/antibodies used.

Antigen	Immunogen	Manufacturer, species, type, catalog number	IHC-TSA+ dilution	IHC-direct dilution	WB dilution	Reference
GFP	recombinant full length protein	Abcam (Cambridge, United Kingdom), chicken polyclonal, Code # ab13970; Lot # 902074	1∶6,000		1∶3,200	[67]
Oestrogen receptor α	KLH-conjugated synthetic peptide representing the last 15 amino acids of rat oestrogen receptor α (TYYIPPEAEGFPNTI)	Millipore (Billerica, MA), rabbit polyclonal, Code # 06-935; Lot # NG1838275		1∶400	1∶4,000	[38]–[40]
Oestrogen receptor β	KLH-conjugated synthetic peptide corresponding to amino acids 63–82 of rat and mouse oestrogen receptor β, and 55–74 of human oestrogen receptor β	Millipore (Billerica, MA), purified rabbit monoclonal, Code # 05-824; Lot # JBC1850147		1∶400	1∶4,000	[40], [41]
Androgen receptor	KLH-conjugated synthetic peptide corresponding to amino acids 1–21 (MEVQLGLGRVYPRPPSKTYRGC) of the human androgen receptor	Millipore (Billerica, MA), affinity purified rabbit polyclonal, Code # 06-680; Lot # DAM1661059		1∶500	1∶4,000	[42], [43]
Calbindin	calbindin D-28k purified from chicken gut	Swant (Bellinzona, Switzerland), mouse monoclonal, Code # 300; Lot No. 07 (F)		1∶1000		[69], [70]
Synaptophysin	synaptosome preparation from rat retina	Sigma-Aldrich (St. Louis, MO), mouse monoclonal, Code # S 5768		1∶100		[71], [72]

### Immunohistochemistry

#### Tissue preparation

All animals were deeply anaesthetised using pentobarbitone sodium (Lethabarb, Virbac, Milperra, NSW, Australia, 100 mg/kg i.p.) and perfused through the heart via the ascending aorta with 20 ml Ca^2+^-free Tyrode's buffer (37°C), followed by 20 ml of a mixture of 4% paraformaldehyde (Sigma-Aldrich, St. Louis, MO) and 0.2% picric acid (Sigma) diluted in 0.16 M phosphate buffer (pH 6.9, 37°C) [65], [66] and 50 ml of the same fixative at 4°C, the latter for approximately 5 min. The brains were dissected out and postfixed in the same fixative for 90 min at 4°C, and finally immersed for 48 h at 4°C in 10% sucrose dissolved in phosphate buffered saline (PBS, pH 7.4) containing 0.01% sodium azide (Sigma) and 0.02% bacitracin (Sigma), before rapid freezing by CO_2_. Sections were cut using a cryostat (Leica CM1850, Wetzlar, Germany) at a thickness of 14 microns, and thaw-mounted on slides coated with 0.5% gelatin- (Sigma) and 0.05% chromium(III) potassium sulphate dodecahydrate (Merck, KGaA, Darmstadt, Germany).

#### Incubation protocol

Sections were rinsed (3×10 min) in 0.01 M PBS and incubated for 24 h at 4°C with the chicken anti-GFP antibody (1∶6,000; Abcam, Cambridge, United Kingdom) diluted in 0.01 M PBS containing 0.3% Triton X-100 and 0.5% BSA (Table 4). To visualize the immunoreactivity, sections were processed using a commercial kit (TSA+, NEN Life Science Products, Inc., Boston, MA). Briefly, sections were washed in TNT buffer (0.1 M Tris-HCl, pH 7.5; 0.15 M NaCl; 0.05% Tween 20, Sigma) for 20 min, incubated with TNB buffer (0.1 M Tris-HCl, pH 7.5; 0.15 M NaCl; 0.5% blocking reagent) for 45 min at room temperature (RT) and incubated with a donkey anti-chicken/horse-radish peroxidase conjugate (Jackson ImmunoResearch Laboratories, West Grove, PA) diluted 1∶500 in TNB buffer for 30 min. Sections were then washed four times in TNT buffer and incubated in a biotinyl tyramide-fluorescein (BT-FITC) conjugate (NEN) diluted 1∶100 in amplification diluent for 10 min at RT. Sections were washed 3×10 min in TNT and 2×10 min in 0.01 M PBS and coverslipped using a fluorescent mounting medium (Dako, Glostrup, Denmark).

For double-immunofluorescence experiments, the immunohistochemical procedure for the EGFP was first completed, followed by incubation for 24 h at 4°C with a rabbit anti-ERα (1∶400; Millipore, Billerica, MA), rabbit anti-ERβ (1∶400; Millipore), rabbit anti-AR (1∶500; Millipore), mouse anti-calbindin (1∶1000; Swant, Bellinzona, Switzerland) or mouse anti-synaptophysin (1∶100; Sigma) antibody (Table 4). Following incubations in: 1) ERα, ERβ or AR antisera; or 2) calbindin or synaptophysin antisera, sections were incubated at RT with Alexa Fluor 555-conjugated donkey anti-rabbit (1∶300, Invitrogen, Eugene, OR) or Alexa Fluor 594-conjugated donkey anti-mouse (1∶300, Invitrogen), respectively, in TNB for 120 min.

### Primary antisera used

#### Enhanced Green Fluorescent Protein

Chicken anti-green fluorescent protein (GFP) polyclonal antibody was raised against the recombinant full length protein (Abcam, Cambridge, United Kingdom; Code No. ab13970; Lot No. 902074; Table 4). Immunoblotting recognises a single band of approximately 27–30 kDa (Abcam), and the antibody did not immunolabel brains of wild-type mice which do not have the EGFP transgene (data not shown). Western blot analysis of mouse brain extracts revealed a single band of approximately 30 kDa. Staining patterns obtained using this antibody were consistent with those previously reported [67].

#### Oestrogen receptor α

Rabbit anti-oestrogen receptor α (C1355) polyclonal antibody, raised against a KLH-conjugated synthetic peptide representing the last 15 amino acids (TYYIPPEAEEGFPNTI) of rat ERα (Millipore, Billerica, MA; Code No. 06-935; Lot No. NG1838275; Table 4). Western blot analysis of mouse brain extracts revealed a single band of approximately 66 kDa, the size of the rat ERα (Fig. 15). The staining patterns obtained using this antibody were consistent with those previously reported in rat [38] and mouse [39], [40].

#### Oestrogen receptor β

Rabbit anti-oestrogen receptor β, clone 68-4 protein A purified monoclonal antibody, raised against a KLH-conjugated synthetic peptide corresponding to amino acids 63–82 of rat and mouse ERβ, and 55–74 of human ERβ (Millipore; Code No. 05-824; Lot No. JBC1850147; Table 4). Western blot analysis of mouse brain extracts revealed a single band of approximately 59 kDa, the size of the rat ERβ (Fig. 15). The staining patterns obtained using this antibody were consistent with those previously reported in mouse [40], [41], and were similar to patterns reported by Shughrue et al. [68], whose antibody did not stain the brain of mice lacking ERβ.

#### Androgen receptor

Rabbit anti-androgen receptor, PG-21 affinity purified polyclonal antibody, raised against a KLH-conjugated synthetic peptide corresponding to amino acids 1–21 (MEVQLGLGRVYPRPPSKTYRGC) of the human AR (Millipore; Code No. 06-680; Lot No. DAM1661059; Table 4). Western blot analysis of mouse brain extracts revealed a single band of approximately 110 kDa, the size of the rat AR (Fig. 15). The staining patterns obtained using this antibody were consistent with those previously reported in mouse [42], [43].

#### Calbindin

Mouse anti-calbindin D-28k monoclonal antibody, raised against calbindin D-28k purified from chicken gut [Swant, Bellinzona, Switzerland; Code No. 300; Lot No. 07 (F); Table 4]. Immunoblotting recognizes a single band of 27–28 KDa, and the antibody does not stain the brain of calbindin D-28k KO mice. The staining pattern of this antibody is consistent with results described previously [69], [70].

#### Synaptophysin

Mouse anti-synaptophysin monoclonal antibody (clone SVP-38), derived from the hybridoma produced by the fusion of mouse myeloma cells and splenocytes, and using a synaptosome preparation from rat retina as the immunogen (Sigma; Code No. S 5768; Table 4). Immunoblotting recognises a single band of approximately 38 kDa (Sigma) [71], [72].

### Image Processing

After processing, sections were examined using a Leica DM LB2 fluorescence microscope (Leica, Wetzlar, Germany), equipped with a dark field condenser and epi-polarization, and epifluorescence with appropriate filter combinations, and with objective lenses of ×10 (N.A. 0.45), ×20 (N.A. 0.70), ×40 (N.A. 0.75), and ×60 oil (N.A. 1.40), and ×100 oil (N.A. 1.30). Photographs were taken using a Hamamatsu ORCA-R^2^ digital camera attached to the Leica DM LB2 microscope, using Hamamatsu HCImage-Live software (Hamamatsu Photonics K.K., Hamamatsu City, Japan). For confocal analysis, an Olympus FV1000 confocal laser scanning microscope (Olympus, Tokyo, Japan) equipped with ×10 (N.A. 0.4), ×20 (N.A. 0.75), ×40 oil (N.A. 1.30) and ×60 oil (N.A. 1.35) objectives was used. The FITC labelling was excited using the 473 nm diode laser. For the detection of AlexaFluor 555, a 559 nm diode laser was used. Z-stack images were captured with multiple images, each separated by a stepwise depth of 0.8–1.0 um in the z-plane.

Digital images from the microscopy were slightly modified to optimise for image resolution, brightness and contrast using Adobe Photoshop CS5 extended, version 12.0, software (Adobe Systems Inc., San Jose, CA), so as to best represent the immunohistochemistry observed at the microscope.

### Cell Quantification

Estimates of the number of EGFP-positive (+), ERα immunoreactive (-ir), ERβ-ir and AR-ir cell bodies in the bed nucleus of the stria terminalis (BST) were obtained from four 14 µm-thick sections, each 280 µm apart, ranging from Bregma +0.38 mm to −0.46 mm [73]. Components of the BST from which these cell bodies were quantified included its: lateral division – dorsal, intermediate, posterior and ventral parts; and medial division – anterior, posterointermediate, posterolateral, posteromedial and ventral parts. Estimates were made using a fractionator sampling design according to optical disector rules [74]–[78], with guard zones of 1 µm (top) and 1 µm (bottom). Regular predetermined intervals (x  =  150 µm, y = 175 µm) and counting frame dimensions (50×50 = 2500 µm^2^) were derived by means of a grid program (Stereoinvestigator v.7.0, MicroBrightField, Williston, VT, viewed through a microscope and ×60 oil objective, Leica). The total number of cell bodies in the BST was estimated by multiplying the number of cell bodies counted within the sampling regions with reciprocals of the fraction of sections sampled, fraction of sectional area sampled, and the fraction of the section thickness sampled.

EGFP-positive (+), ERα-ir, ERβ-ir and AR-ir cell bodies were also quantified in the medial amygdaloid nucleus – posterodorsal (MePD) and posteroventral (MePV) parts. Regions of the MePD/MePV from which cell bodies were quantified corresponded to Bregma −1.34 mm to −2.18 mm [73]. Estimates were made according to the procedure outlined for the BST above, except that regular predetermined intervals of x = 125 µm and y = 150 µm were employed.

All comparisons were conducted by student t-tests and a value of P≤0.05 was considered statistically significant. Values are expressed as the mean ± SEM.

### Data Analysis

The presence of EGFP-like immunoreactivity (-LI) in cell bodies and nerve fibres throughout the mouse brain is summarised in Table 1. EGFP-LI in fibres was described as (+) low, (++) moderate, (+++) high, or (++++) very high density/intensity [79]. The density of EGFP-immunoreactive/positive (+) cell bodies is indicated as (#) sparse, (##) low, (###) medium, or (####) high. The Paxinos and Franklin Stereotaxic Atlas of the Mouse Brain [73] was used to define and label the specific regions expressing EGFP. In Figs. 3–5, 7–9 and 11–13, the stereotaxic co-ordinates indicating distance from Bregma are used only as a guide, and are given to allow the reader to refer to the Atlas mentioned above.
